# The Effect of Dietary Supplements on Oxidative Stress in Pregnant Women with Gestational Diabetes Mellitus: A Network Meta-Analysis

**DOI:** 10.3390/nu13072284

**Published:** 2021-06-30

**Authors:** Christos Chatzakis, Alexandros Sotiriadis, Evangelia Tsakmaki, Maria Papagianni, George Paltoglou, Konstantinos Dinas, George Mastorakos

**Affiliations:** 1 2nd Department of Obstetrics and Gynecology, School of Medicine, Aristotle University of Thessaloniki, 54641 Thessaloniki, Greece; cchatzakis@gmail.com (C.C.); asotir@gmail.com (A.S.); eva.tsakmaki9@gmail.com (E.T.); konstantinosdinas@hotmail.com (K.D.); 2 Endocrine Unit of 3rd Pediatric Department, School of Medicine, Aristotle University of Thessaloniki, 54641 Thessaloniki, Greece; marpapagianni@hotmail.com; 3 Endocrine Unit of Aretaieion Hospital, Medical School, National and Capodistrian University of Athens, 11528 Athens, Greece; gpaltoglou@gmail.com

**Keywords:** gestational diabetes mellitus, oxidative stress, dietary supplement, pregnancy, network meta-analysis

## Abstract

Background: Gestational diabetes mellitus (GDM) exacerbates the oxidative stress status of the pregnant women. Τo improve the oxidative stress status, several therapeutic interventions have been suggested. The aim of this network meta-analysis is to assess the effect of different dietary supplements on the oxidative stress status in pregnant women with GDM. Methods: A network meta-analysis of randomized control trials was performed comparing the changes delta (Δ) in total antioxidant capacity (TAC) and concentration of malondialdehyde (MDA) as primary outcomes, following different therapeutic interventions with dietary supplements in pregnant women with GDM. Four electronic databases and grey literature sources were searched. The secondary outcomes were other markers of oxidative stress. Results: The meta-analysis included 16 studies of 1173 women with GDM. Regarding ΔTAC: probiotics and omega-3 with vitamin E were superior to placebo/no intervention. Regarding ΔMDA: vitamin D with calcium, omega-3, vitamin D, omega-3 with vitamin E, magnesium with zinc and calcium, and probiotics were superior to placebo/no intervention. Conclusions: Administration of dietary supplements in women with GDM can be helpful in limiting the oxidative stress which develop in these pregnancies.

## 1. Introduction

Gestational diabetes mellitus (GDM) is defined as any degree of glucose intolerance with onset or first recognition during the second or third trimester of pregnancy excluding cases of clearly overt diabetes [1]. It is estimated that 17 million or 13.2% of live births to women in 2019 were affected from gestational diabetes mellitus (GDM) [2]. Despite the abundance of management strategies against diabetes, there is still need for both effective and harmless treatments in GDM.

Oxidative stress is defined as an imbalanced equilibrium between the production of reactive oxygen species (ROS) and antioxidant defenses [3]. Increased levels of free radicals and lipid peroxides constitute a normal phenomenon during pregnancy [4,5]. However, excessive oxidation is reported in pregnancies complicated with GDM, mainly due to hyperglycaemia. Excess glucose is responsible for increased free radical production by activating several metabolic mechanisms, such as the polyol pathway, formation of AGE, activation of protein kinase C (PKC), the hexosamine pathway and directly by encouraging the ROS production in the placental mitochondria [3,4,6,7]. Increased levels of oxidative stress are associated with impaired insulin-dependent glucose uptake, elevated apoptosis rate and placental dysfunction, while creating a pro-inflammatory status in general [3]. Therefore, antioxidant supplementation could be beneficial in women with GDM.

Nutritional modifications and diet supplements have been widely tested for their antioxidant capacity. Results in this field are still ambiguous, although the utilization of nutritional supplements in order to ameliorate the oxidation status in non-pregnant populations, is already described in the literature [8,9]. Under this perspective, this network metanalysis aims to assess the effect of different diet supplements on the oxidative stress status in pregnant women with GDM.

## 2. Materials and Methods

### 2.1. Reporting Guideline and Registration

This meta-analysis was performed in compliance with the PRISMA extension statement for network meta-analyses [10] and is registered with PROSPERO (CRD42020160433).

### 2.2. Inclusion Criteria

Randomized controlled trials (RCTs) comparing the effect of dietary supplements (containing probiotics, vitamin D, vitamin E, vitamin C, zinc, soy, omega-3, selenium, magnesium or calcium, alone or combination of them) vs. placebo or nothing on the oxidative stress status of women with GDM were eligible for inclusion. Oxidative markers, such as total antioxidant capacity (TAC), malondialdehyde (MDA) and glutathione (GSH), were considered representative of the oxidative status. No study was excluded due to country or publication date restrictions.

### 2.3. Exclusion Criteria

Studies focused on women with pre-gestational diabetes were excluded.

### 2.4. Primary Outcome Measures

Changes delta (Δ) in the total antioxidant capacity (TAC) and concentrations of malondialdehyde (MDA) from beginning till completion of studies in women receiving either dietary supplements or placebo/no intervention.

### 2.5. Secondary Outcome Measures

Concentrations of TAC, MDA and reduced glutathione (GSH) at completion of studies and ΔGSH concentration from beginning till completion of studies in women receiving either dietary supplements or placebo/no intervention.

### 2.6. Search Methods 

Eligible studies were identified through a pre-defined search strategy in electronic databases (PubMed, Scopus, Cochrane Central Register of Controlled Trials (Central), US Registry of clinical trials (www.clinicaltrials.gov)), using combinations of the terms “pregnancy”, “gestational diabetes mellitus”, “oxidative stress”, “free radicals”, “antioxidants” “dietary supplements”, “probiotics”, “vitamin D”, “vitamin E”, “vitamin C”, “zinc”, “soy”, “omega-3”, “selenium”, “magnesium” or “calcium”. The references of the retrieved articles and additional automated search using PubMed’s “search for related articles” function was used complementary to the main search. Duplicate or overlapping samples were excluded after deliberate data comparison. In the case of overlap, the study with the largest number of cases was included.

### 2.7. Study Selection

Two reviewers (CC and ET) assessed independently the eligibility of all identified studies according to the aforementioned criteria. Disagreements between reviewers were reconciled by arbitration of a third reviewer (AS).

### 2.8. Data Extraction

Information from each study was extracted independently by two reviewers (CC and ET) using a predefined data extraction form. Disagreements were resolved by discussion between the two review authors until consensus is reached.

### 2.9. Risk of Bias

Included studies were objectively assessed for internal validity using the Cohrane “Risk of bias” tool 2 [11]. Five distinct domains are assessed to detect bias arising from randomization, deviations from intended interventions, missing outcome data, outcome measurements and in selection the reported result. Studies were classified as being of “low risk” when risk of bias was low for all domains. Studies were classified as “concern of bias” when one domain was rated as “some concern”. Studies were classified as “high risk” when at least one domain was rated as “high risk” or multiple domains were rated as “concern of bias”.

### 2.10. Geometry of the Networks

A network plot was constructed for each of the outcomes (TAC, ΔMDA, TAC, MDA, ΔGSH, and GSH), including all groups which received either a dietary supplement or placebo/no intervention. All groups were represented by nodes and head-to-head comparisons with edges. The size of a node was proportional to the number of patients; width of the edges was proportional to the number of trials evaluating each intervention (dietary supplement or placebo/no intervention); the color of each edge was matched to the average risk of bias for each head-to-head comparison. Network plots were constructed using the “networkplot” command of the “network graphs” package in Stata (Stata 15.1, Statacorp LP, College Station, TX, USA) [12].

### 2.11. Assessment of Transitivity

Transitivity, a fundamental assumption of network meta-analysis, implies that two interventions can be validly compared via a connected indirect route involving one or more intermediate comparators. Transitivity can be evaluated by comparing the distribution of the potential effect modifiers across the available direct comparisons in the network [13]. Details on patient and study characteristics that could act as effect modifiers were recorded. Furthermore, in order to statistically assess transitivity, we performed a meta-analysis for the baseline values of the primary outcomes TAC and MDA in intervention (any) and control/no intervention groups and then we proceeded with a leave one out analysis to investigate the influence of each individual study on the overall meta-analysis summary estimate.

### 2.12. Statistical Analysis

When direct comparisons were available, a standard random-effects meta-analysis was initially performed initially for the outcomes. Direct estimates were derived using a comparison-specific random-effects model on Open Meta-Analyst (http://www.cebm.brown.edu/openmeta/, accessed on 1 November 2019). A random-effects network meta-analysis was performed subsequently, to compare simultaneously the relative effectiveness of all interventions [14]. A common heterogeneity (τ) across all comparisons was assumed and compared with previously derived empirical distributions for heterogeneity [15]. For all possible pairwise comparisons, mean difference (MD) or standardized mean difference (SMD) with 95% confidence interval (CI) were estimated using the multivariate meta-analysis approach in which the different comparisons in studies are treated as different outcomes accounting for the correlation introduced by multi-arm trials [16]. The network meta-analysis models were performed using the “network” package in Stata. Analyses were performed as per intention to treat. Prediction intervals (PrIs), which indicate the interval within which the relative effect of a future study is expected to lie, were estimated and plotted to aid interpretation of the random-effects network meta-analysis [17]. This was accomplished using the “network graphs” package in Stata. The PrI plot provides information about the extent and impact of the common heterogeneity on each relative intervention effect [18]. For each intervention, the ranking probabilities of assuming any possible rank was estimated, by plotting the cumulative ranking curves and calculating the surface under them (surface under the cumulative ranking, SUCRA). The latter serves in percentage form expressing the effectiveness of an intervention compared to a theoretical intervention which is always the best without uncertainty. The larger the SUCRA value, the better the rank of the intervention [18]. Contribution plots were constructed to assess the influence of every direct comparison to each network estimate and the entire network [18].

### 2.13. Assessment of Inconsistency

An inconsistency plot was designed using the “ifplot” command in Stata to evaluate the consistency of intervention effects (i.e., the agreement between direct and indirect evidence. In each loop, the inconsistency factor was estimated as the ratio of the two mean differences or standardized mean differences from direct and indirect evidence for one comparison in the loop. Statistical agreement between two different sources of evidence is represented by values close to those shown in [18]. Significant inconsistency in a loop was detected in case the unity was not included in the 95% CI diagnosed. We performed this approach assuming a common heterogeneity parameter across all loops in the network as this was estimated from the network meta-analysis model.

### 2.14. Assessment of Small-Study Effects

The effect of small studies, proxying order to refrain from publication bias, was assessed using a comparison-adjusted funnel plot, which accounted for the estimate effects of the studies for different comparisons across the network [18].

## 3. Results

### 3.1. Search Results

**Figure 1:**
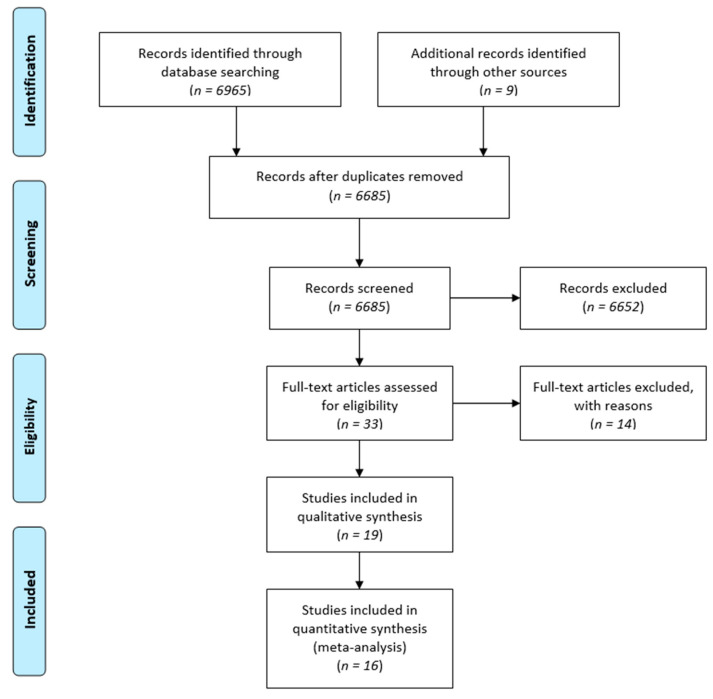
Flow chart of the different phases of the systematic review.

The electronic search initially retrieved 6974 records. The flowchart of the selection procedure is presented in Figure 1. After exclusion with reasons of 14 studies, 19 were included in the qualitative synthesis (characteristics of the included studies are presented in Table 1). The 14 excluded studies along with the reason of their exclusion are presented in Table S1. Three studies were included in the systematic review but not in the quantitative synthesis because one did not provide necessary descriptive statistic measures [19], and in the two other studies the outcome measures did not match the predefined primary and secondary outcomes [20,21]. Finally, 16 studies of 1173 women with GDM, were included in the quantitative synthesis (meta-analysis).

### 3.2. Geometry of the Networks

**Figure 2:**
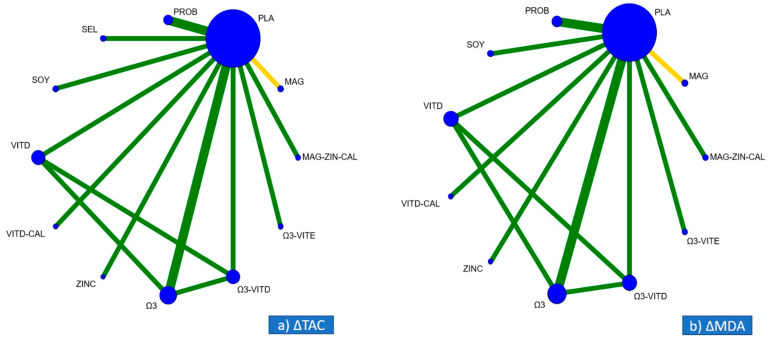
Network plots for the primary outcomes of (**a**) ΔTAC (changes in total antioxidant capacity) and (**b**) ΔMDA (changes in malondialdehyde). Interventions are represented by nodes and head-to-head comparisons with edges. The size of the nodes is proportional to the number of the patients, while the thickness of the edges is proportional to the number of studies. The color of the edges represents the average risk of bias for each head-to-head comparison, green for low risk of bias, yellow for uncertain risk of bias, and red for high risk of bias.

The network plot for changes in TAC is presented in Figure 2a. All interventions were pairwise tested in 12 studies. The most common comparison was probiotic supplementation vs. placebo/no intervention (two studies) [25,28]. The most studied patients were included in probiotic supplementation vs. placebo (120 patients, two studies) [25,28], Vitamin D vs. no intervention (114 patients, two studies) [22,33] and Omega-3 vs. no intervention (114 patients, two studies) [31,33] Placebo/no intervention was given in 361 patients (12 studies) [23,25,28,29,30,31,32,33,34,35,36].

The network plot for the changes in the MDA is presented in Figure 2b. All interventions were pairwise tested in 10 studies. The most common comparison was probiotic supplementation vs. no intervention (two studies) [25,28]. The most studied patients were included in probiotics vs. placebo (120 patients, two studies) [25,28] and Omega-*3* versus placebo (114 patients, two studies) [31,33]. Placebo/no intervention was given in 299 patients (ten studies) [23,25,28,29,30,31,32,33,35,36].

### 3.3. Risk of Bias

The risk of bias is presented in Table 2. The randomization procedure was not described in one study [37]. The exercise study used a diet modification as intervention (soy protein as part of total protein), therefore blindness was not possible [35]. However, the effect of lack of blinding of the assessors is likely to be limited, as the outcomes (effects on metabolic profile, inflammatory factors and biomarkers of oxidative stress) are objectively defined and assessed following the predefined strategy, and therefore are less susceptible to bias. Overall, one out of 16 studies was of “some concerns” of bias [37].

### 3.4. Assessment of Transitivity and Inconsistency

No discrepancies in study and participant characteristics or the definition of interventions and outcomes were detected among studies which compared more than one dietary supplements (Table 1). Transitivity was examined by assessing inconsistency. No such inconsistency was detected.

Furthermore, the result of the leave one out analysis, showed that there was no study diverting from the average estimate (Figure S1).

### 3.5. Primary Outcomes of the Meta-Analysis Regarding ΔTAC and ΔMDA

**Figure 3:**
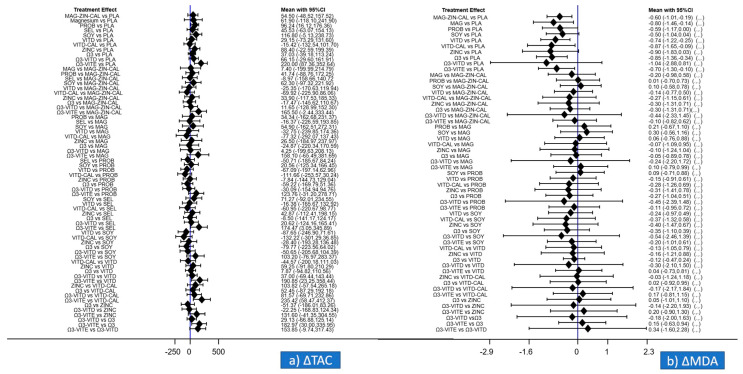
Mean difference (MD) for (**a**) ΔTAC (changes in total antioxidant capacity) and (**b**) ΔMDA (changes in malondialdehyde) as estimated from the network meta-analysis for every possible pair of interventions. Solid lines represent 95% Confidence Intervals (CIs).

**Figure 4:**
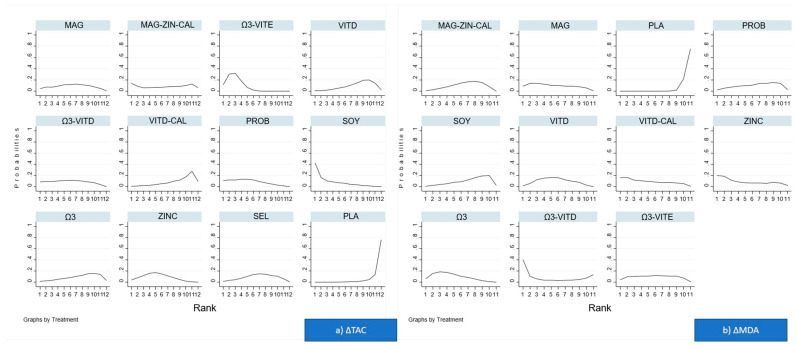
Rankograms for (**a**) ΔTAC (changes in total antioxidant capacity) and (**b**) ΔMDA (changes in malondialdehyde) and the surface under the cumulative ranking curve (SUCRA) for each intervention. Horizontal axis shows possible ranks and vertical axis shows probability that an intervention is at each rank.

Regarding ΔTAC: among all supplements administrated, probiotics (MD: 96.24; 95% CI: 16.12–176.36) and omega-3 with vitamin E (MD:220.0; 95% CI 87.36–352.64, respectively) were superior to placebo/no intervention, and (Figure 3a, Table 3). Regarding relative ranking, among all supplements administrated, omega-3 with vitamin E had the highest SUCRA value (95.5%), followed by soy (72.3%) and probiotics (67.2%), while placebo/no intervention was the least effective (Figure 4a).

Regarding ΔMDA: among all supplements administrated, vitamin D with calcium (MD: −0.87; 95% CI −1.65–−0.09), omega-3 (MD: −0.85; 95% CI: −1.36–−0.34), magnesium (MD: −0.80; 95% CI: −1.46–−0.14), vitamin D (MD: −0.74; 95% CI: −1.22–−0.25), omega-3 with vitamin E (MD: −0.70; 95% CI: −1.3–−0.10), magnesium with zinc and calcium (MD: −0.60; 95% CI: −1.01–−0.19), and probiotics (MD: −0.59; 95% CI: −1.17–−0.0) were superior to placebo/no intervention (Figure 3b, Table 4). Regarding relative ranking: among all supplements administrated omega-3 had the highest SUCRA value (66.6%), closely followed by zinc (65.2%), while placebo/no intervention was the least effective (Figure 4b).

### 3.6. Secondary Outcomes of the Meta-Analysis Regarding TAC, GSH, MDA, ΔGSH 

Regarding TAC concentrations (Figure S2a): among all supplements administrated either vitamin C (MD −2.87, 95% CI −3.06 to −2.68), or vitamin D (MD −1.29, 95% CI −1.78 to −0.79), or omega-3 with vitamin D (MD −1.29, 95% CI −1.78 to −0.79), or omega-3 with vitamin E (MD −1.00, 95% CI −1.67 to −0.33), or probiotics (MD −0.78, 95% CI −0.98 to −0.57), or magnesium with zinc and calcium (MD −0.70, 95% CI −1.17 to −0.23), or omega-3 (MD −0.59, 95% CI −1.07 to −0.10) or probiotics with vitamin D (MD −0.36, 95% CI −0.70 to −0.02) were superior compared to placebo/no intervention (Figure S3a, Table 5). In terms of relative ranking, omega-3 with vitamin E had the highest SUCRA value (77.3%), followed by omega-3 with vitamin D (73.6%) and probiotics (69.3%); vitamin D with calcium were the least effective (Table S2).

Regarding MDA concentrations (Figure S2b): among all supplements administrated either vitamin D, or omega-3 with vitamin D, or probiotics or omega-3 were superior to placebo/no intervention (MD −2.10, 95% CI −2.86 to −1.35, MD −1.72, 95% CI −2.73 to −0.72, MD −0.76, 95% CI −1.32 to −0.20 and MD −0.98, 95% CI −1.81 to −0.14, respectively) (Figure S3b, Table 6). In terms of relative ranking, vitamin C and had the highest SUCRA value (100%), followed by vitamin D (83.7) and omega-3 with vitamin D (83.7%); placebo/no intervention was the least effective (Table S2).

Regarding ΔGSH (Figure S2c), all supplements administrated vitamin D with calcium, or soya, or selenium, or omega-3 with vitamin D or probiotics were superior to placebo/no intervention (MD 98.4, 95% CI 8.6 to 188.2, MD 94.6, 95% CI 31.7 to 157.4, MD 92.0, 95% CI 37.6 to 146.4 MD 80.2, 95% CI 30.8 to 129.7 and MD 40.7, 95% CI 10.1 to 71.3, respectively) (Figure S3c, Table 7). In terms of relative ranking, soya had the greatest SUCRA value (85%), followed by selenium (84.4%) and vitamin D with calcium (83.4%); magnesium was the least effective (Table S2).

Regarding GSH concentrations (Figure S2d): among all supplements administrated vitamin C was superior to placebo/no intervention (MD 3.42, 95% CI 2.37 to 4.48) (Figure S3d, Table 8). In terms of relative ranking, vitamin C had the highest SUCRA value (97.8%), followed by omega-3 with vitamin D (82.9%) and probiotics (62.8%); soya was the least effective (Table S2).

### 3.7. Small-Study Effects

**Figure 5:**
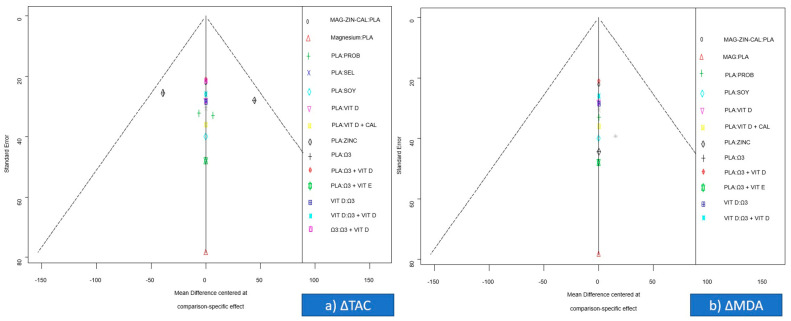
Comparison adjusted funnel plot of the included studies for the primary outcomes of (**a**) ΔTAC (changes in total antioxidant capacity) and (**b**) ΔMDA (changes in malondialdehyde).

Comparison-adjusted funnel plots were symmetric for all outcomes, indicating a lack of significant small study effect (Figure 5).

### 3.8. Quality of the Evidence

The overall quality of the evidence as per GRADE (Grading of Recommendations Assessment, Development and Evaluation) criteria adapted for network meta-analysis was moderate to high and is presented in detail in Tables S3 and S4 [38].

Study limitations: the contributions of direct and indirect data to the network estimate are presented in Figure S4. The evidence in all network estimates was downgraded (a) by one level, if >50% of the information came from studies at “some concerns” or “high risk” of bias, (b) by two levels, if >50% of the information came from studies at “high risk” of bias.

Indirectness: There were no differences in the baseline patient characteristics (including the BMI at randomization) among the studies. Thus, no downgrading took place.

Inconsistency: Due to the lack of closed loops, the point estimates of direct and indirect comparisons were evaluated. The evidence was downgraded by one level, if the prediction intervals extended across the line of no effect.

Imprecision: The evidence was downgraded by one level, if the prediction intervals extended across the line of no effect.

Publication bias: The comparison-adjusted funnel plot was symmetric for all outcomes (Figure 5). Thus, no downgrading took place.

## 4. Discussion

### 4.1. Summary of the Evidence

Following analysis from 16 RCTs which studied pregnant women with (1173) GDM, we found that several dietary supplements are superior to placebo/no intervention regarding improvement of oxidative stress status in women with GDM. The majority of dietary supplements studied led to decrease and increase in concentrations of pro- and anti- oxidation biomarkers, respectively.

### 4.2. Interpretation

In normal pregnancy, the metabolic adaptations and secretion of placental hormones leads to the development of “physiological” insulin resistance, which, ultimately, serves to ensuring the transfer of the appropriate amounts of glucose to the fetus [39,40]. Gestational diabetes mellitus develops in women whose pancreatic function is insufficient to overcome this pregnancy-associated insulin resistance, resulting in increased concentrations of blood glucose [41]. The hyperglycemic environment in GDM is strongly associated with the emergence of oxidative stress, which is characterized by increased and decreased concentrations of pro- and anti-oxidation biomarkers [42,43,44,45,46,47,48,49,50]. Increased blood glucose concentrations lead to interaction of blood glucose with proteins, forming advanced glycation end-products (AGEs) [51,52,53,54]. This modification of proteins alters their function [55,56,57,58]. Extracellular AGEs bind to the receptor of AGEs (RAGE) and activate different intracellular signaling molecules such as nuclear factor-kappa B (NF-kB), an intra-cytoplasmic molecule whose activation leads to the formation of reactive oxygen species (ROS) via NADPH oxidase activity [3,59] Furthermore, increased blood glucose concentrations result in lipid peroxidation. The resulting products damage cell membranes and lead to the production of end-products such as MDA [60,61]. In addition, increased blood glucose concentrations activate the hexosamine biosynthetic pathway to produce glucosamine-6-phosphate, an inhibitor of the glucose-6-phosphate dehydrogenase (G6PD), which holds a crucial role in the pentose phosphate pathway [62,63,64,65]. The latter produces an important amount of the NADPH in cells. Activated G6PD converts glucose-6-phosphate into glucose-6-phosphogluconate and, subsequently, via formation of NADPH to ribose-5-phosphate. Therefore, inhibition of G6PD will result in decreased production of NADPH [66]. The latter is instrumental in anti-oxidation due to its action as electron donor in the reduction in glutathione disulfide (GSSG) to glutathione (GSH), which reduces free hydrogen peroxides to water (Figure S5) [67,68,69,70].

Omega-3 fatty acids either alone or in combination with other interventions, are consistently score in the first most effective interventions for the primary outcomes and for the majority of the secondary outcomes. Administration of omega-3 fatty acids in pregnant women with GDM is justified by their capacity to improve the oxidative status in various ways. Previous studies have shown that administration of omega-3 fatty acids increase the expression of peroxisome proliferator-activated receptor gamma (PPAR-γ) resulting thus, to inhibition of NF-kB activation and subsequent secretion of proinflammatory cytokines [71,72,73]. In addition, administration of omega-3 fatty acids leads to the increase in glutathione concentrations, an antioxidant [74,75,76]. Moreover, omega-3 fatty acids prevent oxidation of plasma lipids [77,78,79]. Indeed, in this metanalysis we found that administration of omega-3 fatty acids was superior to placebo/no intervention regarding the increase and decrease in GSH and MDA, respectively.

Similarly, administration of probiotics in pregnant women with GDM is justified by their capacity to attenuate the negative effects of oxidative stress. Their administration results in production of metabolites with anti-oxidation capacity such as glutathione (GSH) [80]. In addition, probiotics increase expression of PPAR-γ which inhibits activation of NF-kB and secretion of proinflammatory cytokines [81,82]. Furthermore, probiotics prevent oxidation of plasma lipids [80,83]. Indeed, in this metanalysis we have shown that administration of probiotics was superior to placebo/no intervention regarding production of GSH and MDA.

Vitamin E and vitamin C exert their anti-oxidation capacity either by reducing or by preventing oxidative damage. Vitamin E prevents lipid peroxidation chain reactions in cellular membranes by interfering with propagation of lipid radicals. Vitamin C even in small amounts, can protect proteins, lipids, carbohydrates, and nucleic acids from damage due to pro-oxidants generated physiologically. Vitamin C is also responsible for restoration of oxidized glutathione (GSSG) back to its reduced isoform GSH [84,85,86]. Administration of vitamin D has been shown to decrease peroxidation of lipids, production of AGEs and to increase activity of glutathione enzymes [87,88,89].

### 4.3. Strengths and Limitations

This is the first metanalysis assessing the effect of different dietary supplements upon pro- or anti-oxidation status of women with GDM. The present network metanalysis provides a global comparison and ranking of these interventions. Network metanalysis allows assessment of dietary supplements not directly compared in the same study [90,91] and ranks the compared interventions to identify the optimal one, based on their probability to be the most effective, as expressed by the SUCRA values [12] (References [92,93,94,95,96,97,98,99,100,101,102,103] are cited in the supplementary materials). Furthermore, this is the first study providing, via GRADE, an overview of the quality of the current evidence for the dietary interventions in women with GDM. This methodological approach evaluates evidence according to combinations of outcomes and comparisons. The moderate-to-high quality evidence analyzed in this metanalysis is translated into moderate-to-high confidence in the results adding to the strengths of the study. An inherent limitation of any network metanalysis is that ranking can be misleading, if interpreted “as it is”. The assessment of the differences in the effect estimates among ranks should consider the clinical implications of the ranking difference between two interventions. In addition, a potential limitation of the present network meta-analysis is that there is no correlation between the improvement of oxidative stress status in women with GDM and clinical maternal and fetal/neonatal outcomes. However, the clinical outcomes are not in the scope of the present study and an additional study which will address this issue may follow.

## 5. Conclusions

In conclusion, this network metanalysis provides convincing evidence that the use of dietary supplements in women with GDM can be helpful in limiting the deleterious oxidative effects which develop in these pregnancies.

## Figures and Tables

**Table 1:** Characteristics of the included studies.

	Author	Country	Participants	Intervention	Intervention Period	Outcomes
**VITAMIN D**						
1	Asemi 2013 [22]	Iran	54 pregnant women diagnosed with GDM (27 vitamin D, 27 Placebo)	Oral vitamin D3 50,000 IU, 2 times in 6 wk (1 at baseline, 2nd at day 21)	6 weeks (from 24–28 wk of gestation)	FPG, HOMA-IR, HOMA-B, QUICKI, plasma TAC, GSH, total cholesterol, LDL cholesterol, HDL cholesterol, serum calcium, triglycerides, hs-CRP, Insulin
2	Zhang 2016 [19]	China	133 pregnant women diagnosed with GDM (38 200 IU vitamin D, 38 50,000 IU monthly (2000 IU daily for 25 days), 37 50,000 IU every 2 weeks (4000 IU daily for 12.5 days), 20 Placebo)	Oral 200 IU vitamin D or 50,000 IU monthly (2000 IU daily for 25 days) or 50,000 IU every 2 weeks (4000 IU daily for 12.5 days)	From 24–28 weeks of pregnancy until delivery	FPG, Insulin, HOMA-IR, total cholesterol, triglycerides, hs-CRP, TAC, GSH
**VITD + CALCIUM**						
3	Asemi 2014 [23]	Iran	56 pregnant women diagnosed with GDM (28 Calcium-Vitamin D, 28 Placebo)	Oral 1000 mg calcium per day and Vitamin D3 50,000 IU 2 times in 6 wk (1 at baseline, 2nd at day 21)	6 weeks (from 24–28 wk of gestation)	FPG, HOMA-IR, HOMA-8, Insulin, QUICKI, Total Cholesterol, Triacylglycerol, LDL cholesterol, HDL cholesterol, hs-CRP, NO, TAC, GSH, MDA
**VITD + PROBIOTIC/PROBIOTIC ALONE**						
4	Jamilian 2018 [24]	Iran	87 pregnant women diagnosed with GDM (30 Vitamin D plus Probiotic, 29 Probiotic, 28 Placebo)	Oral 50,000 IU vitamin D3 every 2 weeks plus 8 × 10^9^ CFU/g probiotic containing *L. acidophilus*, *B. bifidum, L. reuteri, and L fermentum* (each 2 × 10^9^) or 8 × 10^9^ CFU/g probiotic containing *Lactobacillus acidophilus, Bifidobacterium bifidum, L. reuteri, and Lactobacillus fermentum* (each 2 × 10^9^)	6 weeks (from 24–28 wk of gestation)	25-hydroxyvitamin D, FPG, Insulin, HOMA-IR, QUICKI, Triglycerides, VLDL-cholesterol, total cholesterol, LDL-cholesterol, HDL-cholesterol, total-/HDL-cholesterol ratio, hs-CRP, NO, TAC, GSH, MDA, newborns’ hospitalization, newborns’ hyperbilirubinemia, polyhydramnios, preterm delivery, newborns’ macrosomia > 4000 g, c-section, gestational age, pre-eclampsia, newborns’ weight, newborns’ length, newborns’ head circumference, Apgar score, newborns’ hypoglycemia
**PROBIOTICS**						
5	Karamali 2018 [25]	Iran	60 pregnant women diagnosed with GDM (30 synbiotic capsule, 30 Placebo)	One oral synbiotic capsule containing *Lactobacillus acidophilus* strain T16, *L. casei* strain T2 and *Bifidobacterium bifidum* strain T1 (2 × 10^9^ CFU/g each) plus 800 mg inulin	6 weeks	Primary outcomes were inflammatory markers (hs-CRP). The secondary outcomes were biomarkers of oxidative stress (TAC, NO, GSH, MDA) and pregnancy outcomes (c-section, preterm delivery, pre-eclampsia, polyhydramnios, maternal hospitalization, macrosomia > 4000 g, gestational age, newborns’ weight, newborns’ length, newborns’ head circumference, Apgar score, newborns’ hyperbilirubinemia, newborns’ hypoglycemia)
6	Babadi 2018 [26]	Iran	48 pregnant women diagnosed with GDM (24 Probiotic supplement, 24 Placebo)	Oral probiotic capsule containing *Lactobacillus acidophilus, Lactobacillus casei, Bifidobacterium bifidum, and Lactobacillus fermentum* (2 × 10^9^ CFU/g each) per day	6 weeks (from 24–28 wk of gestation)	Gene expression of PPAR-γ, TGF-β, VEGF and TNF-α, LDRL, IL-1, IL-8 FPG, serum insulin, HOMA-IR, QUICKI, triglycerides, VLDL-cholesterol, total-/HDL-cholesterol ratio, HDL cholesterol, MDA, NO, TAC
7	Hajifaraji 2018 [27]	Iran	64 pregnant women diagnosed with GDM (32 probiotic supplement, 32 Placebo). Finally, 56 analyzed (29 probiotic, 27 placebo)	Oral probiotic capsule containing *L. acidophilus LA-5, Bifidobacterium BB-12, Streptococcus thermophilus and Lactobacillus delbrueckii bulgaricus* (4 biocap > 4 × 10^9^ CFU) per day	8 weeks (from 24 to 28-weeks (+6 days) of gestation)	hs-CRP, TNF-α, IL-6, TAC, MDA, serum GSHR, erythrocyte GPx, serum uric acid, erythrocyte SOD
8	Badehnoosh 2018 [28]	Iran	60 pregnant women diagnosed with GDM (30 Probiotic supplement, 30 Placebo)	Oral probiotic capsule containing *Lactobacillus acidophilus, Lactobacillus casei and Bifidobacterium bifidum* (2×10^9^ CFU/g each) per day	6 weeks (from 24–28 wk of gestation)	FPG, hs-CRP, NO, TAC, GSH, MDA, MDA/TAC, c-section, preterm delivery, need to insulin therapy after intervention, pre-eclampsia, polyhydramnios, maternal hospitalization, macrosomia > 4000 g, gestational age, newborn’s weight, newborn’s length, newborn’s head circumference, LGA, Apgar score (1 min and 5 min), newborns’ hyperbilirubinemia, newborn’s hospitalization, newborn’s hypoglycemia
**MAGNESIUM**						
9	Asemi 2015 [29]	Iran	70 pregnant women diagnosed with GDM (35 Magnesium, 35 Placebo)	Oral magnesium supplement 250 mg daily for 6 wk	6 weeks (from 24–28 wk of gestation)	FPG, HOMA-IR, QUICKI, serum insulin levels, hs-CRP, plasma GSH, triglycerides, HOMA-B, MDA, plasma NO, TAC, GSH, magnesium, total cholesterol, newborn hyperbilirubinemia, newborn hospitalization rate, c-section, need for insulin therapy, polyhydramnios, maternal hospitalization, preterm delivery, gestational age, newborn birth size, Apgar score, and newborn hypoglycemia, macrosomia, preterm delivery, preeclampsia, weight-length-head circumference of newborns
**MAGN-ZINC-CALCIUM-VITD**						
10	Jamilian 2019 [30]	Iran	60 pregnant women diagnosed with GDM (30 magnesium-zinc-calcium-vitamin D co-supplementation, 30 Placebo)	Oral 100 mg magnesium, 4 mg zinc, 400 mg calcium plus 200IU vitamin D supplements twice a day	6 weeks	hs-CRP, FPG, Magnesium, Zinc, Calcium, 25-OH-vitamin D, total nitrite, TAC, GSH, MDA, c-section, preterm delivery, need to insulin therapy after intervention, pre-eclampsia, polyhydramnios, macrosomia > 4000 g, gestational age, newborns’ weight, newborns’ length, newborns’ head circumference, Apgar score, newborns’ hyperbilirubinemia, newborns’ hypoglycemia
**OMEGA-3**						
11	Jamilian 2016 [31]	Iran	54 pregnant women diagnosed with GDM (27 omega-3 supplement, 27 Placebo)	Oral 1000 mg omega-3 fatty acid supplements (containing 180 mg eicosapentaenoic acid and 120 mg docosahexanoic acid) per day	6 weeks (from 24–28 wk of gestation)	maternal polyhydramnios, pre-eclampsia, gestational age, cesarean section, newborn’s size, Apgar score, hyperbilirubinemia, inflammatory factors and biomarkers of oxidative stress (hs-CRP, TAC, NO, GSH, MDA)
**OMEGA-3 + VIT E**						
12	Jamilian 2017 [32]	Iran	60 pregnant women diagnosed with GDM (30 Omega-3 plus Vitamin E supplements, 30 Placebo)	Oral 1000 mg omega-3 fatty acids from flaxseed oil plus 400 IU vitamin E supplements per day	6 weeks	TAC, NO, GSH, MDA, hs-CRP, maternal polyhydramnios, preeclampsia, gestational age, caesarean section, newborn’s size, Apgar score, newborns’ hyperbilirubinemia, newborns’ hospitalization
**OMEGA-3 + VIT D**						
13	Razavi 2017 [33]	Iran	120 pregnant women diagnosed with GDM (30 omega-3, 30 vitD, 30 omega-3 +vitD, 30 Placebo)	1000 mg omega-3 fatty acids containing 180 mg eicosapentaenoic acid (EPA) and 120 mg docosahexaenoic acid (DHA) twice a day or 50,000 IU vitamin D every 2 weeks or both	6 weeks (from 24–28 wk of Gestation)	Primary outcome: inflammatory factors (hs-CRP) Secondary outcomes: biomarkers of oxidative stress (TAC, NO, GSH, MDA) and pregnancy outcomes (c-section, preterm delivery, pre-eclampsia, polyhydramnios, macrosomia > 4000 g, gestational age, newborns’ weight, newborns’ length, newborns’ head circumference, Apgar score, newborns’ hyperbilirubinemia, newborns’ hospitalization, newborns’ hypoglycemia)
**SELENIUM**						
14	Asemi 2015 [34]	Iran	70 pregnant women diagnosed with GDM (35 Selenium, 35 Placebo)	Oral selenium supplement 200 µg as tablet per day for 6 wk	6 weeks (from 24–28 wk of Gestation)	FPG, HOMA-IR, QUICKI, serum insulin levels, hs-CRP, plasma GSH, plasma MDA, HOMA-B, lipid profiles, plasma NO, TAC concentrations, systolic and diastolic blood pressure, c-section, newborn’s hyperbilirubinemia, weight-height-head circumference of newborns
**Alpha-Lipoid-Acid**						
15	Aslfalah 2019 [21]	Iran	60 pregnant women diagnosed with GDM (30 Alpha-lipoic acid (ALA), 30 Placebo)	Orally one capsule of ALA (100 mg) per day for 8 weeks during lunch	8 weeks (from 24–28 wk of gestation)	FPG, Insulin, HOMA-IR, QUICKI, ALA, Adiponectin, Leptin, MDA/TAC, A/L ratio, L/A ratio, A/H ratio
**SOY**						
16	Fei 2014 [20]	China	97 pregnant women diagnosed with GDM (46 SBOS, 51 Placebo)	SBOS (soybean oligosaccharides) 10 g/day in 200–300 mL warm water, took in orally before sleep). 100 min in week 2.	8 weeks	SOD, Catalase, GPx, TBARS, FPG, FINS, Adiponectin, HOMA-IR, HBCI (islet b-cells function index), need to insulin therapy after intervention
17	Jamilian 2015 [35]	Iran	68 pregnant women diagnosed with GDM (34 soy protein, 34 Placebo)	A diet (soy diet) containing the 0.8-g/kg protein with 35% animal protein, 35% soy protein, and 30% other plant proteins	6 weeks	FPG, Insulin, HOMA-IR, HOMA-B, QUICKI, triglycerides, VLDL-C, TC, LDL cholesterol, HDL cholesterol T-/HDL-C ratio, hs-CRP, NO, TAC, GSH, MDA, newborn hyperbilirubinemia, newborn hospitalization, c-section, need for insulin therapy after the intervention, polyhydramnios, maternal hospitalization, preterm delivery, gestational age, newborn birth size, Apgar score, and newborn hypoglycemia
**ZINC**						
18	Karamali 2015 [36]	Iran	50 pregnant women diagnosed with GDM (25 zinc supplementation, 25 Placebo)	Oral 233 mg zinc gluconate (containing 30 mg zinc)	6 weeks (from 24–28 wk of Gestation)	Zinc, hs-CRP, TAC, MDA, NO, GSH, c-section, need to insulin therapy after intervention, polyhydramnios, macrosomia > 4000 g, gestational age, newborns’ weight, newborns’ length, newborns’ head circumference, Apgar score, newborns’ hyperbilirubinemia, newborns’ hypoglycemia
**VITAMIN C**						
19	Maged 2016 [37]	Egypt	200 pregnant women diagnosed with GDM (100Vitamin C, 100 Placebo)	Orally 1-g L-ascorbic acid (vitamin C) per day	from 28–32 weeks until the time of delivery	Neonatal outcomes (Apgar score, neonatal sugar, NICU admission, RDS, hypoglycemia, hyperbilirubinemia needing phototherapy, hyperbilirubinemia needing exchange transfusion, perinatal mortality) nonenzymatic (GSH and MDA) and enzymatic (SOD, CAT, GPx) oxidative stress parameters in placental tissues homogenates, maternal blood plasma/lysate and neonatal blood.

A/H ratio: Adiponectin to HOMA-IR ratio; A/L ratio: Adiponectin to Leptin ratio; FINS: Fasting Insulin; FPG: Fasting Plasma Glucose; GDM: Gestational Diabetes Mellitus; GP: Glutathione Peroxidase; GSH: Glutathione; GSHR: Glutathione Reductase; HDL: High-density Lipoprotein; HOMA-B: Homeostatic Model Assessment for Beta cell function; HOMA-IR: Homeostatic Model Assessment for Insulin Resistance; hs-CRP: High-sensitivity C-reactive Protein; IL-1: Interleukin-1; IL-8: Interleukin-8; L/A ratio: Leptin to Adiponectin ratio; LDL: Low-density Lipoprotein; LDRL: Low-density Lipoprotein Receptor; LGA: Large for Gestational Age; MDA: Malondialdehyde; NICU: Neonatal Intensive Care Unit; NO: Nitric Oxide; PPAR-γ: Peroxisome Proliferator Activated Receptor gamma; QUICKI: Quantitative Insulin Sensitivity Check Index; RDS: Respiratory Distress Syndrome; SOD: Superoxide Dismutase; TAC: Total Antioxidant Capacity; TBAR: Thiobarbituric Acid Reactive Substances; TC: Total Cholesterol; TGF-β: Transforming Growth Factor beta; TNF-α: Tumor Necrosis Factor alpha; VEGF: Vascular Endothelial Growth Factor; VLDL: Very-low-density Lipoprotein.

**Table 2:** Risk of bias of selected studies.

Study	Intervention	Randomization Process	Deviations from Intended Interventions	Missing Outcome Data	Measurement of the Outcome	Selection of the Reported Results	Overall
Asemi 2013 [22]	Vitamin D	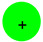	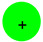	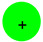	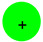	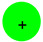	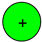
Asemi 2014 [23]	Vitamin D + calcium	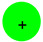	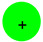	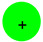	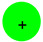	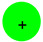	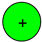
Asemi 2015 [34]	Selenium	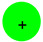	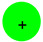	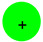	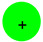	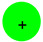	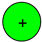
Asemi 2015 [29]	Magnesium	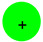	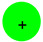	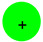	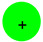	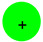	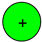
Badehnoosh 2018 [28]	Probiotics	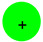	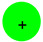	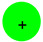	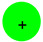	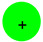	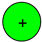
Jamilian 2015 [35]	Soy	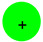	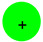	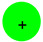	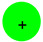	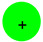	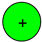
Jamilian 2016 [31]	Omega3	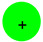	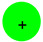	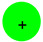	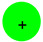	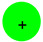	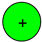
Jamilian 2017 [32]	Omega3 + Vitamin E	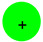	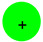	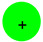	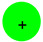	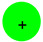	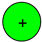
Jamilian 2019 [30]	magnesium-zinc-calcium-vitamin D	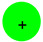	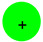	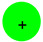	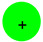	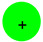	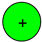
Karamali 2015 [36]	Zinc	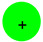	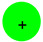	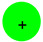	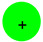	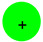	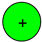
Karamali 2018 [25]	Probiotics	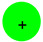	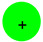	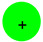	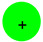	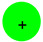	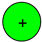
Razavi 2017 [33]	Vitamin D + Omega3	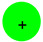	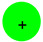	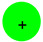	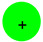	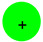	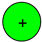
Babadi 2018 [26]	Probiotics	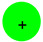	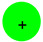	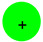	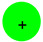	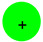	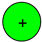
Hajifaraji 2018 [27]	Probiotics	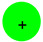	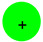	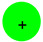	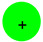	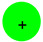	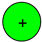
Jamilian 2018 [24]	Vitamin D + Probiotics	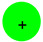	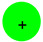	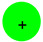	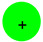	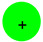	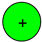
Zhang 2016 [19]	Vitamin D	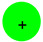	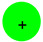	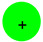	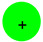	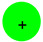	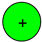
Maged 2016 [37]	Vitamin C	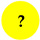	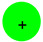	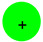	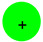	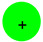	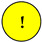
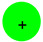 Low risk 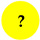 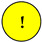 Some concerns 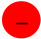 High risk							

**Table 3:** Direct and indirect (in grey) estimates of the mean differences of ΔTAC. Each column presents a different intervention.

MAG+ZINC+CAL											54.50 (11.27, 97.73)
7.40 (−199.99, 214.79)	MAG										61.90 (−91.90, 215.70)
41.74 (−88.76, 172.25)	34.34 (−162.68, 231.37)	PROB									96.34 (51.10, 141.58)
−8.97 (−158, 66, 140, 72)	−16.37 (−226.59, 193.85)	−50.71 (−185.67, 84.24)	SEL								45.53 (−9.70, 100.76)
62.30 (−97.32, 221.92)	54.90 (−162.51, 272.31)	20.56 (−125.34, 166.45)	71.27 (−92.01, 234.55)	SOY							116.80 (38.55, 195.05)
−25.35 (−170.63, 119.94)	−32.75 (−239.85, 174.36)	−67.09 (−197.14, 62.96)	−16.38 (−165.67, 132.92)	−87.65 (−246.90, 71.61)	VIT D			−33.30 (−89.13, 22.53)	37.00 (−13.85, 87.85)		14.85 (−81.08, 110.79)
−69.92 (−225.90, 86.06)	−77.32 (−292.07, 137.43)	−111.66 (−253.57, 30.24)	−60.95 (−220.67, 98.77)	−132.22 (−301.29, 36.85)	−44.57 (−200.18, 111.03)	VIT D + CAL					−15.42 (−85.95, 55.11)
33.90 (−117.53, 185.33)	26.50 (−184.97, 237.97)	−7.84 (−144.73, 129.04)	42.87 (−112.41, 198.15)	−28.40 (−193.28, 136.48)	59.25 (−91.80, 210.29)	103.82 (−57.54, 265.18)	ZINC				88.40 (28.61, 148.19)
−17.47 (−145.62, 110.67)	−24.87 (−220.34, 170.59)	−59.22 (−169.79, 51.36)	−8.50 (−141.17, 124.17)	−79.77 (−223.56, 64.02)	7.87 (−94.82, 110.56)	52.45 (−87.29, 192.18)	−51.37 (−186.01, 83.26)	Ω3	3.70 (−38.61, 46.01)		35.68 (−68.64, 140.01)
11.65 (−128.99, 152.30)	4.25 (−199.63, 208.13)	−30.09 (−154.94, 94.76)	20.62 (−124.16, 165.41)	−50.65 (−205.68, 104.39)	37.00 (−69.44, 143.44)	81.57 (−69.71, 232.86)	−22.25 (−168.83, 124.34)	29.13 (−66.88, 125.14)	Ω3 + VIT D		91.10 (50.05, 132.15)
165.50 (−2.44, 333.44)	158.10 (−65.49, 381.69)	123.76 (−31.20, 278.71)	174.47 (3.05, 345.89)	103.20 (−76.97, 283.37)	190.85 (23.25, 358.44)	235.42 (58.47, 412.37)	131.60 (−41.35, 304.55)	182.97 (30.00, 335.95)	153.85 (−9.74, 317.43)	Ω3 + VIT E	220.00 (125.93, 314.07)
54.5 (−48.52, 157.52)	61.9 (−118.1, 241.9)	96.24 (16.12, 176.36)	45.53 (−63.07, 154.13)	116.8 (−5.13, 238.73)	29.15 (−73.3, 131.6)	−15.42 (−132.54, 101.7)	88.40 (−22.6, 199.4)	37.03 (−39.18, 113.24)	66.15 (−29.6, 161.91)	220.0 (87.36, 35)	PLA

ΔTAC: changes in Total Antioxidant Capacity; Ω3: Omega-3 fatty acids; CAL: Calcium; MAG: Magnesium; PLA: Placebo; PROB: Probiotics; SEL: Selenium; VIT D: Vitamin D.

**Table 4:** Direct and indirect (in grey) estimates of the mean difference of ΔMDA. Each column presents a different intervention.

MAG+ZINC+CAL										−0.60 (−1.01, −0.19)
−0.20 (−0.98, 0.58)	MAG									−0.80 (−1.46, −0.14)
0.01 (−0.70, 0.73)	0.21 (−0.67, 1.10)	PROB								−0.45 (−0.74, −0.16)
0.10 (−0.58, 0.78)	0.30 (−0.56, 1.16)	0.09 (−0.71, 0.88)	SOY							−0.50 (−1.04, 0.04)
−0.14 (−0.77, 0.50)	0.06 (−0.76, 0.88)	−0.15 (−0.91, 0.61)	−0.24 (−0.97, 0.49)	VIT D			0.10 (−0.27, 0.47)	−0.30 (−0.55, −0.05)		−0.70 (−1.23, −0.17)
−0.27 (−1.15, 0.61)	−0.07 (−1.09, 0.95)	−0.28 (−1.26, 0.69)	−0.37 (−1.32, 0.58)	−0.13 (−1.05, 0.79)	VIT D + CAL					−0.87 (−1.65, −0.09)
−0.30 (−1.31, 0.71)	−0.10 (−1.24, 1.04)	−0.31 (−1.41, 0.78)	−0.40 (−1.47, 0.67)	−0.16 (−1.21, 0.88)	−0.03 (−1.24, 1.18)	ZINC				−0.90 (−1.83, 0.03)
−0.30 (−1.31, 0.71)	−0.05 (−0.89, 0.78)	−0.27 (−1.04, 0.51)	−0.35 (−1.10, 0.39)	−0.12 (−0.47, 0.24)	0.02 (−0.92, 0.95)	0.05 (−1.01, 1.10)	Ω3	−0.20 (−0.57, 0.17)		−0.85 (−1.36, −0.34)
−0.44 (−2.33, 1.45)	−0.24 (−2.20, 1.72)	−0.45 (−2.39, 1.48)	−0.54 (−2.46, 1.39)	−0.30 (−2.10, 1.50)	−0.17 (−2.17, 1.84)	−0.14 (−2.20, 1.93)	−0.18 (−2.00, 1.63)	Ω3 + VIT D		−1.00 (−1.53, −0.47)
−0.10 (−0.82, 0.62)	0.10 (−0.79, 0.99)	−0.11 (−0.95, 0.72)	−0.20 (−1.01, 0.61)	0.04 (−0.73, 0.81)	0.17 (−0.81, 1.15)	0.20 (−0.90, 1.30)	0.15 (−0.63, 0.94)	0.34 (−1.60, 2.28)	Ω3 + VIT E	−0.70 (−1.30, −0.10)
−0.60 (−1.01, −0.19)	−0.80 (−1.46, −0.14)	−0.59 (−1.17, 0.00)	−0.50 (−1.04, 0.04)	−0.74 (−1.22, −0.25)	−0.87 (−1.65, −0.09)	−0.90 (−1.83, 0.03)	−0.85 (−1.36, −0.34)	−1.04 (−2.88, 0.81)	−0.70 (−1.30, −0.10)	PLA

ΔMDA: changes in Malondialdehyde; Ω3: Omega-3 fatty acids; CAL: Calcium; MAG: Magnesium; PLA: Placebo; PROB: Probiotics; SEL: Selenium; VITnD: Vitamin D.

**Table 5:** Direct and indirect (in grey) estimates of the mean difference of TAC. Each column presents a different intervention.

MAG+ZINC+CAL												−20.80 (−68.41, 26.81)
266.23 (−18.84, 551.31)	VIT D + PROB											156.00 (108.10, 203.90)
76.10 (−219.56, 371.76)	190.13 (−133.69, 513.96)	MAG										55.30 (−105.51, 216.11)
145.63 (−63.27, 354.54)	120.60 (−73.37, 314.57)	69.53 (−189.76, 328.83)	PROB									102.07 (30.83, 173.30)
91.70 (−165.40, 348.80)	−174.53 (−463.58, 114.51)	15.60 (−283.89, 315.09)	−53.93 (−268.23, 160.36)	SEL								70.92 (3.47, 138.37)
24.30 (−236.11, 284.71)	−241.93 (−533.93, 50.06)	−51.80 (−354.13, 250.53)	−121.33 (−339.59, 96.92)	−67.40 (−332.15, 197.35)	SOY							3.50 (−75.62, 82.62)
92.98 (−131.05, 317.01)	−173.26 (−433.30, 86.78)	16.88 (−254.75, 288.51)	−52.66 (−225.85, 120.54)	1.28 (−227.78, 230.34)	68.68 (−164.09, 301.45)	VIT D			−37.10 (−89.16, 14.96)	136.20 (92.10, 180.30)		110.77 (66.44, 155.10)
−17.25 (−278.25, 243.75)	−283.48 (−576.00, 9.03)	−93.35 (−396.19, 209.49	−162.88 (−381.84, 56.07)	−108.95 (−374.28, 156.38)	−41.55 (−310.08, 226.98)	−110.23 (−343.65, 123.20)	VIT D + CAL					−38.05 (−119.08, 42.98)
37.00 (−222.19, 296.19)	−229.23 (−520.14, 61.67)	−39.10 (−340.38, 262.18)	−108.63 (−325.43, 108.16)	−54.70 (−318.25, 208.85)	12.70 (−254.08, 279.48)	−55.98 (−287.38, 175.43)	98.44 (−128.89, 325.77)	ZINC				16.20 (−58.80, 91.20)
81.19 (−136.48, 298.86)	−185.05 (−439.85, 69.76)	5.09 (−261.32, 271.50)	−64.45 (−229.68, 100.78)	−10.51 (−233.36, 212.33)	56.89 (−169.77, 283.54)	−11.79 (−162.61, 139.02)	242.74 (−6.52, 492.00)	44.19 (−181.06, 269.44)	Ω3	99.10 (49.18, 149.02)		54.10 (−137.46, 245.66)
225.49 (−14.99, 465.97)	−40.74 (−315.13, 233.64)	149.39 (−135.96, 434.74)	79.86 (−114.21, 273.92)	133.79 (−111.39, 378.97)	201.19 (−47.45, 449.83)	132.51 (−36.19, 301.22)	−18.13 (−120.65, 84.39)	188.49 (−58.88, 435.86)	144.30 (−20.15, 308.76)	Ω3 + VIT D		249.40 (205.49, 293.31)
358.70 (95.98, 621.42)	92.47 (−201.59, 386.52)	282.60 (−21.73, 586.93)	213.07 (−7.94, 434.08)	267.00 (−0.02, 534.02)	334.40 (64.19, 604.61)	265.72 (30.37, 501.08)	−69.61 (−182.38, 43.16)	321.70 (52.66, 590.74)	277.51 (48.20, 506.82)	133.21 (−117.86, 384.28)	Ω3 + VIT ET E	337.30 (250.85, 423.75)
−20.80 (−199.43, 157.83)	245.43 (23.27, 467.60)	55.30 (−180.29, 290.89)	124.83 (16.52, 233.15)	70.90 (−114.00, 255.80)	3.50 (−185.98, 192.98)	72.18 (−63.03, 207.38)	−38.05 (−228.34, 152.24)	16.20 (−171.60, 204.00)	60.39 (−63.99, 184.76)	204.69 (43.69, 365.69)	337.90 (145.25, 530.55)	PLA

Ω3: Omega-3 fatty acids; CAL: Calcium; MAG: Magnesium; PLA: Placebo; PROB: Probiotics; SEL: Selenium; TAC: Total Antioxidant Capacity; VIT D: Vitamin D.

**Table 6:** Direct and indirect (in grey) estimates of the mean difference of MDA. Each column presents a different intervention.

MAG+ZINC+CAL												−0.70 (−1.17, −0.23)
0.34 (−0.24, 0.92)	VIT D + PROB											−0.30 (−0.74, 0.14)
0.50 (−0.35, 1.35)	−0.16 (−0.94, 0.62)	MAG										−0.20 (−0.90, 0.50)
−0.08 (−0.59, 0.44)	0.42 (0.11, 0.72)	−0.58 (−1.31, 0.16)	PROB									−0.78 (−0.98, −0.57)
0.10 (−0.74, 0.94)	0.24 (−0.53, 1.01)	−0.40 (−1.39, 0.59)	0.18 (−0.55, 0.90)	SOY								−0.60 (−1.29, 0.09)
−2.17 (−2.68, −1.66)	2.51 (2.12, 2.90)	−2.67 (−3.40, −1.94)	−2.09 (−2.37, −1.82)	−2.27 (−2.99, −1.55)	VIT C							−2.87 (−3.06, −2.68)
−0.59 (−1.27, 0.10)	0.93 (0.33, 1.53)	−1.09 (−1.95, −0.23)	−0.51 (−1.05, 0.02)	−0.69 (−1.54, 0.16)	1.58 (1.05, 2.11)	VIT D			−0.70 (−0.88, −0.52)	0.00 (−0.15, 0.15)		−1.20 (−1.78, −0.62)
0.13 (−0.74, 1.00)	0.21 (−0.59, 1.01)	−0.37 (−1.38, 0.64)	0.21 (−0.55, 0.96)	0.03 (−0.98, 1.04)	2.30 (1.55, 3.05)	0.72 (−0.16, 1.60)	VIT D + CAL					−0.57 (−1.30, 0.16)
0.90 (−0.26, 2.06)	0.56 (−0.55, 1.67)	0.40 (−0.87, 1.67)	0.98 (−0.10, 2.05)	0.80 (−0.47, 2.07)	3.07 (1.99, 4.15)	1.49 (0.32, 2.66)	0.77 (−0.52, 2.06)	ZINC				0.20 (−0.86, 1.26)
0.11 (−0.56, 0.79)	−0.23 (−0.82, 0.36)	−0.39 (−1.24, 0.47)	0.19 (−0.34, 0.71)	0.01 (−0.83, 0.86)	2.28 (1.76, 2.80)	0.70 (0.59, 0.81)	−0.02 (−0.89, 0.86)	−0.79 (−1.95, 0.38)	Ω3	−0.70 (−0.88, −0.52)		−0.59 (−1.08, −0.10)
−0.59 (−1.27, 0.10)	−0.93 (−1.53, −0.33)	−1.09 (−1.95, −0.23)	−0.51 (−1.05, 0.02)	−0.69 (−1.54, 0.16)	1.58 (1.05, 2.11)	0.00 (−0.15, 0.15)	−0.72 (−1.60, 0.16)	−1.49 (−2.66, −0.32)	−0.70 (−0.81, −0.59)	Ω3 + VIT D		−1.20 (−1.78, −0.62)
−0.30 (−1.12, 0.52)	−0.64 (−1.39, 0.11))	−0.80 (−1.77, 0.17)	−0.22 (−0.92, 0.47)	−0.40 (−1.36, 0.56)	1.87 (1.18, 2.56)	0.29 (−0.54, 1.12)	−0.43 (−1.42, 0.56)	−1.20 (−2.45, 0.05)	−0.41 (−1.24, 0.41)	0.29 (−0.54, 1.12)	Ω3 + VIT E	−1.00 (−1.67, −0.33)
−0.70 (−1.17, −0.23)	−0.36 (−0.70, −0.02)	−0.20 (−0.90, 0.50)	−0.78 (−0.98, −0.57)	−0.60 (−1.29, 0.09)	2.87 (−3.06, −2.68)	−1.29 (−1.78, −0.79)	−0.57 (−1.30, 0.16)	0.20 (−0.86, 1.26)	−0.59 (−1.07, −0.10)	−1.29 (−1.78, −0.79)	−1.00 (−1.67, −0.33	PLA

Ω3: Omega-3 fatty acids; CAL: Calcium; MAG: Magnesium; MDA: Malondihaldehyde; PLA: Placebo; PROB: Probiotics; SEL: Selenium; VIT D: Vitamin D.

**Table 7:** Direct and indirect (in grey) estimates of the mean difference of ΔGSH. Each column presents a different intervention.

MAG+ZINC+CAL											20.90 (−19.17, 60.97)
71.17 (2.80, 139.54)	SEL										92.07 (37.66, 146.48)
−99.00 (−195.11, −2.89)	170.17 (67.79, 272.55)	MAG									−78.10 (−164.83, 8.63)
19.82 (−31.66, 71.30)	51.35 (−11.06, 113.77)	118.82 (26.85, 210.79)	PROB								40.72 (10.13, 71.31)
73.70 (−1.58, 148.98)	2.53 (−80.61, 85.67)	172.70 (65.58, 279.82)	53.88 (−16.04, 123.80)	SOY							94.60 (31.73, 157.47)
8.04 (−51.61, 67.69)	−63.13 (−132.44, 6.17)	107.04 (10.26, 203.82)	−11.78 (−64.49, 40.94)	−65.66 (−141.79, 10.47)	VIT D			0.70 (0.52, 0.88)	52.90 (14.70, 91.10)		−199.20 (−716.26, 317.85)
77.51 (−21.39, 176.41)	6.34 (−98.67, 111.35)	176.51 (51.65, 301.37)	57.69 (−37.19, 152.57)	3.81 (−105.82, 113.44)	69.47 (−30.08, 169.02)	VIT D + CAL					3.87 (−85.94, 93.68)
−30.80 (−101.42, 39.82)	−101.97 (−180.92, −23.02)	68.20 (−35.70, 172.10)	−50.62 (−115.49, 14.26)	−104.50 (−189.51, −19.49)	−38.84 (−110.37, 32.69)	−108.31 (−214.80, −1.82)	ZINC				−9.90 (−67.11, 47.31)
10.76 (−48.85, 70.37)	−60.41 (−129.69, 8.86)	109.76 (13.00, 206.51)	−9.06 (−61.74, 43.62)	−62.94 (−139.05, 13.16)	2.72 (−29.06, 34.50)	−66.75 (−166.28, 32.77)	41.56 (−29.94, 113.06)	Ω3	45.90 (6.80, 85.00)		26.71 (−18.18, 71.59)
59.38 (−5.11, 123.86)	−11.79 (−85.30, 61.72)	158.38 (58.55, 258.21)	39.56 (−18.57, 97.70)	−14.32 (−94.30, 65.66)	51.34 (13.37, 89.32)	−18.13 (−120.65, 84.39)	90.18 (14.57, 165.79)	48.62 (9.89, 87.35)	Ω3 + VIT D		83.70 (28.82, 138.58)
7.90 (−71.88, 87.68)	−63.27 (−150.51, 23.97)	106.90 (−3.43, 217.23)	−11.92 (−86.66, 62.83)	−65.80 (−158.56, 26.96)	−0.14 (−80.73, 80.45)	−69.61 (−182.38, 43.16)	38.70 (−50.32, 127.72)	−2.86 (−83.42, 77.70)	−51.48 (−135.71, 32.75)	Ω3 + VIT E	28.80 (−39.40, 97.00)
20.90 (−20.51, 62.31)	92.07 (37.66, 146.48)	−78.10 (−164.83, 8.63)	40.72 (10.13, 71.31)	94.60 (31.73, 157.47)	28.94 (−13.99, 71.87)	98.41 (8.60, 188.22)	−9.90 (−67.11, 47.31)	31.66 (−11.23, 74.54)	80.28 (30.84, 129.72)	28.80 (−39.40, 97.00)	PLA

ΔGSH: changes in Glutathione; Ω3: Omega-3 fatty acids; CAL: Calcium; MAG: Magnesium; PLA: Placebo; PROB: Probiotics; SEL: Selenium; VIT D: Vitamin D.

**Table 8:** Direct and indirect (in grey) estimates of the mean difference of GSH. Each column presents a different intervention.

MAG+ZINC+CAL													19.20 (−37.70, 76.10)
−0.45 (−1.97, 1.07)	MAG												−37.90 (−100.12, 24.32)
−0.22 (−1.74, 1.30)	0.23 (−1.28, 1.74)	SEL											−7.97 (−83.12, 67.18)
3.26 (1.74, 4.77)	3.71 (2.21, 5.21)	3.47 (1.97, 4.97)	VIT C										0.76 (0.70, 0.82)
0.08 (−1.13, 1.30)	0.53 (−0.67, 1.73)	−0.30 (−1.50, 0.90)	3.18 (1.99, 4.36)	PROB									28.60 (−10.48, 67.68)
−0.76 (−2.29, 0.77)	−0.31 (−1.82, 1.21)	−0.54 (−2.06, 0.97)	4.01 (2.51, 5.52)	−0.84 (−2.05, 0.37)	SOY								−106.80 (−191.91, −21.69)
−0.54 (−1.85, 0.78)	−0.09 (−1.39, 1.22)	−0.32 (−1.62, 0.98)	−3.79 (−5.08, −2.50)	−0.62 (−1.54, 0.31)	0.22 (−1.09, 1.53)	VIT D				−82.90 (−112.63, −53.17)	114.60 (84.08, 145.12)		−32.46 (−131.25, 66.33)
−0.02 (−1.48, 1.44)	0.43 (−1.02, 1.88)	0.20 (−1.25, 1.65)	−3.28 (−4.71, −1.84)	−0.10 (−1.08, 0.88)	0.74 (−0.72, 2.19)	0.52 (−0.71, 1.75)	VIT D + PROB						−28.60 (−128.80, 71.60)
−0.50 (−2.04, 1.04)	−0.05 (−1.58, 1.48)	−0.28 (−1.81, 1.25)	−3.76 (−5.28, −2.24)	−0.58 (−1.81, 0.64)	0.26 (−1.28, 1.79)	0.04 (−1.29, 1.36)	0.48 (−0.99, 1.95)	VIT D + CAL					−92.37 (−235.91, 51.17)
−0.08 (−1.63, 1.47)	0.37 (−1.17, 1.91)	0.14 (−1.40, 1.68)	−3.34 (−4.87, −1.81)	−0.16 (−1.40, 1.07)	0.68 (−0.87, 2.22)	0.46 (−0.88, 1.79)	−0.06 (−1.54, 1.42)	0.42 (−1.14, 1.98)	ZINC				9.90 (−51.44, 71.24)
−0.02 (−1.33, 1.30)	0.43 (−0.87, 1.74)	0.20 (−1.10, 1.50)	−3.27 (−4.57, −1.98)	−0.10 (−1.03, 0.83)	0.74 (−0.57, 2.05)	0.52 (−0.37, 1.41)	0.00 (−1.23, 1.24)	0.48 (−0.84, 1.81)	0.06 (−1.27, 1.40)	Ω3	31.70 (1.66, 61.74)		−8.45 (−188.50, 171.59)
0.64 (−0.82, 2.10)	1.09 (−0.36, 2.54)	0.86 (−0.59, 2.31)	−2.62 (−4.05, −1.18)	0.56 (−0.56, 1.68)	1.40 (−0.06, 2.85)	1.18 (0.16, 2.20)	0.66 (−0.72, 2.05)	1.14 (−0.33, 2.61)	0.72 (−0.76, 2.20)	0.66 (−0.36, 1.67)	Ω3 + VIT D		110.20 (48.54, 171.86)
−0.45 (−1.99, 1.08)	0.00 (−1.53, 1.52)	−0.24 (−1.76, 1.29)	−3.71 (−5.22, −2.20)	−0.53 (−1.75, 0.68)	0.30 (−1.22, 1.83)	0.08 (−1.23, 1.40)	−0.43 (−1.90, 1.03)	0.05 (−1.49, 1.59)	−0.37 (−1.92, 1.18)	−0.44 (−1.76, 0.88)	−1.09 (−2.56, 0.37)	Ω3 + VIT E	−48.10 (−132.46, 36.26)
0.17 (−0.92, 1.25)	−0.28 (−1.35, 0.79)	−0.05 (−1.12, 1.02)	3.42 (2.37, 4.48)	0.25 (−0.30, 0.80)	−0.59 (−1.66, 0.49)	−0.37 (−1.11, 0.38)	0.15 (−0.83, 1.13)	−0.33 (−1.43, 0.76)	0.09 (−1.02, 1.20)	0.15 (−0.60, 0.90)	0.81 (−0.17, 1.79)	0.28 (−1.37, 0.80)	PLA

Ω3: Omega-3 fatty acids; CAL: Calcium; GSH: Glutathione; MAG: Magnesium; PLA: Placebo; PROB: Probiotics; SEL: Selenium; VIT D: Vitamin D.

## Data Availability

Not applicable.

## Supplementary Materials

The following are available online at https://www.mdpi.com/article/10.3390/nu13072284/s1, Figure S1: Leave one out analysis for the baseline values of A) TAC and B) MDA, investigating the influence of each individual study on the overall meta-analysis summary estimate, Figure S2: Network plots for the secondary outcomes of A) TAC B) MDA, C) ΔGSH, D) GSH. Treatments are represented by nodes and head-to-head comparisons with edges. The size of the nodes is proportional to the number of the patients, while the thickness of the edges is proportional to the number of studies. The color of the edges represents the average risk of bias for each head-to-head comparison, green for low risk of bias, yellow for uncertain risk of bias, and red for high risk of bias, Figure S3: Mean difference (MD) for A) TAC, B) MDA, C) ΔGSH and D) GSH as estimated from the network meta-analysis for every possible pair of interventions. Solid lines represent 95% Confidence Intervals (Cis), Figure S4: The contributions of direct and indirect data to the network estimate, Figure S5: Pathophysiology between hyperglycemia and impairment of antioxidant mechanisms, Table S1: Excluded studies, Table S2: SUCRA values of different antibiotic treatments for the primary and secondary outcomes, Table S3: GRADE for the ΔTAC outcome, Table S4: GRADE for the ΔMDA outcome.
