# Supplementary material for: The Effect of Dietary Supplements on Oxidative Stress in Pregnant Women with Gestational Diabetes Mellitus: A Network Meta-Analysis
Source: Nutrients. 2021 Jun 30;13(7):2284. doi: 10.3390/nu13072284 (PMC8308478; doi:10.3390/nu13072284)
Supplement: Supplementary file 1 [file nutrients-13-02284-s001.zip › nutrients-1213638-supplementary.pdf]

Table S1. Excluded studies.

#	Author	PMID or DOI	Reason for exclusion
1.	Asemi 2013 [92]	23466048	Diet modification (DASH diet) rather than specific dietary supplementation.
2	Jamilian 2018 [73]	29385062	No markers of oxidative stress were assessed
3	Lopez-Tinoco2011 [93]	21327985	Not a randomized clinical trial
4	Samimi 2015 [94]	24973862	No markers of oxidative stress were assessed
5	Smith 2015 [95]	10.1016/j.ajog.2014.10.864	Pharmaceutical intervention (metformin) rather than dietary supplementation.
6	Yazdchi 2016 [96]	27247730	No markers of oxidative stress were assessed
7	Yessoufou 2015 [97]	25961055	Not a randomized clinical trial
8	Aslfalah 2018 [98]	30328246	Same population examined in Aslfalah 2018-adiponectin
9	Dolatkhah 2015 [99]	26825666	No markers of oxidative stress were assessed
10	Li, Q., & Xing, B. 2016 [100]	27336154	No markers of oxidative stress were assessed
11	Valizadeh, M. 2016 [101]	27679649	Outcomes were measured 6-12 weeks post-partum rather than during pregnancy.
12	Liang HY [102]	19620065	Article is written in non-European language (Chinese)
13	Jafarnejad 2016 [103]	27429803	No markers of oxidative stress were assessed
14	Zhang 2016 [19]	27588106	SD of the outcomes was not provided specifically and could only be speculated by the graphs.

Table S2. SUCRA values of different antibiotic treatments for the primary and secondary outcomes.

Rank	SUCRA ΔTAC	SUCRA ΔMDA	SUCRA TAC	SUCRA MDA	SUCRA ΔGSH	SUCRA GSH
**1**	Ω-3 + VIT E (95.5)	Ω-3 (66.5)	Ω-3 + VIT E (96.4)	VIT C (100)	SOY (85)	VIT C (97.8)
**2**	SOY (72.3)	ZINC (65.1)	Ω-3 + VIT D (85.2)	VIT D (83.7)	SEL (84.4)	Ω-3 + VIT D (82.9)
**3**	PROB (67.2)	Ω-3 + VIT D (64.7)	PROB + VITD (84.6)	Ω-3 + VIT D (83.7)	VIT D + CAL (83.4)	PROB (62.8)
**4**	ZINC (62.1)	VIT D + CAL (64.3)	PROB (69.3)	Ω-3 + VIT E (68.8)	Ω-3 + VIT D (80.6)	Ω-3 (56.5)
**5**	Ω-3 + VIT D (53.7)	MAG (60.1)	VIT D (53.1)	PROB (58.3)	PROB (54.2)	MAG-ZIN-CAL (55.5)
**6**	MAG (49.6)	VIT D (54.3)	SEL (51.6)	MAG-ZIN-CAL (52.3)	Ω-3 (46.1)	VIT D + PROB (55.3)
**7**	MAG-ZIN-CAL (47.3)	Ω-3 + VIT E (52.1)	Ω-3 (49.1)	SOY (45.3)	Ω-3 + VIT E (44.5)	ZINC (51.3)
**8**	SEL (43.1)	MAG-ZIN-CAL (42.6)	MAG (47.6)	Ω-3 (44.1)	VIT D (43.7)	PLA (47.3)
**9**	Ω-3 (38.3)	PROB (42.4)	ZINC (36.7)	VIT D + CAL (43.4)	MAG-ZIN-CAL (38.1)	SEL (45.1)
**10**	VIT D (34.8)	SOY (35.1)	SOY (33.2)	PROB + VIT D (28.9)	PLA (19.5)	MAG (33.1)
**11**	VIT D + CAL (18.3)	PLA (2.6)	PLA (27.7)	MAG (22.2)	ZINC (18.6)	Ω-3 + VIT E (32.9)
**12**	PLA (17.3)		MAG-ZIN-CAL (26.4)	ZINC (10.4)	MAG (2.1)	VIT D + CAL (31.7)
**13**			VIT D + CAL (22.7)	PLA (9.0)		VIT D (25.9)
**14**						SOY (19.8)

Table S3. GRADE of the ΔTAC outcome (changes in Total Antioxidant Capacity) outcome

Outcomes	№ of participants (studies)	Mean Difference (95% CI)	Certainty of the evidence (GRADE)	Comments
MAG-ZIN-CALvs Placebo (direct evidence)	60 (1 study)	**54.50 (1.27, 97.73)**	High	
MAG-ZIN-CALvs Placebo (Network MA)		**54.50 (-48.52,157.52)**	Moderate	c
MAG vs Placebo (direct evidence)	70 (1 study)	**61.90 (-91.90, 215.70)**	Low	1,4
MAG vs Placebo (Network MA)		**61.90 (-118.10,241.90)**	Low	a, c
PROB vs Placebo (direct evidence)	120 (2 studies)	**96.34 (51.10, 141.58)**	High	
PROB vs Placebo (Network MA)		**96.24 (16.12,176.36)**	High	
SEL vs Placebo (direct evidence)	70 (1 study)	**45.53 (-9.70, 100.76)**	Moderate	4
SEL vs Placebo (Network MA)		**45.53 (-63.07,154.13)**	Moderate	c
SOY vs Placebo (direct evidence)	68 (1 study)	**116.80 (38.55, 195.05)**	High	
SOY vs Placebo (Network MA)		**116.80 (-5.13,238.73)**	Moderate	c
VIT D vs Placebo (direct evidence)	60 (1 study)	**14.85 (-81.08, 110.79)**	Moderate	4
VIT D vs Placebo (Network MA)		**29.15 (-73.29,131.60)**	Moderate	c
VIT D-CAL vs Placebo (direct evidence)	56 (1 study)	**-15.42 (-85.95, 55.11)**	Moderate	4
VIT D-CAL vs Placebo (Network MA)		**-15.42 (-132.54,101.70)**	Moderate	c
ZINC vs Placebo (direct evidence)	50 (1 study)	**88.40 (28.61, 148.19)**	High	
ZINC vs Placebo (Network MA)		**88.40 (-22.59,199.39)**	Very low	c
Ω3 vs Placebo (direct evidence)	114 (2 studies)	**35.68 (-68.64, 140.01)**	Moderate	4
Ω3 vs Placebo (Network MA)		**37.03 (-39.18,113.24)**	High	
Ω3-VIT D vs Placebo (direct evidence)	60 (1 study)	**91.10 (50.05, 132.15)**	High	
Ω3-VIT D vs Placebo (Network MA)		**66.15 (-29.60,161.91)**	Moderate	c
Ω3-VIT E vs Placebo (direct evidence)	60 (1 study)	**220.00 (125.93, 314.07)**	High	
Ω3-VIT E vs Placebo (Network MA)		**220.00 (87.36,352.64)**	High	
Ω3 vs VIT D (direct evidence)	60 (1 study)	**33.30 (-22.57,89.12)**	Moderate	4
Ω3 vs VIT D (Network MA)		**7.87 (-94.82,110.56)**	Moderate	c
Ω3-VIT D vs VIT D (direct evidence)	60 (1 study)	**37.0 (-13.84,87.84)**	Moderate	4
Ω3-VIT D vs VIT D (Network MA)		**37.00 (-69.44,143.44)**	Moderate	c
Ω3-VIT D vs Ω3 (direct evidence)	60 (1 study)	**3.70 (-38.60,46.0)**	Moderate	4
Ω3-VIT D vs Ω3 (Network MA)		**29.13 (-66.88,125.14)**	Moderate	c
MAG-ZIN-CALvs MAG (Network MA)		**7.40 (-199.99, 214.79)**	Moderate	c

Outcomes	№ of participants (studies)	Mean Difference (95% CI)	Certainty of the evidence (GRADE)	Comments
MAG-ZIN-CALvs PROB (Network MA)		**41.74 (-88.76, 172.25)**	Moderate	c
MAG-ZIN-CALvs SEL (Network MA)		**-7.40 (-199.99, 214.79)**	Moderate	c
MAG-ZIN-CALvs SOY (Network MA)		**62.30 (-97.32, 221.92)**	Moderate	c
MAG-ZIN-CALvs VIT D (Network MA)		**-25.35 (-170.63, 119.94)**	Moderate	c
MAG-ZIN-CALvs VIT D - CAL (Network MA)		**--25.35 (-170.63, 119.94)**	Moderate	c
MAG-ZIN-CALvs ZINC (Network MA)		**33.90 (-117.53, 185.33)**	Moderate	c
MAG-ZIN-CALvs Ω3 (Network MA)		**-17.47 (-145.62, 110.67)**	Moderate	c
MAG-ZIN-CALvs Ω3 – VIT D (Network MA)		**11.65 (-128.99, 152.30)**	Moderate	c
MAG-ZIN-CALvs Ω3 – VIT E (Network MA)		**165.50 (-2.44, 333.44)**	Moderate	c
MAG vs PROB (Network MA)		**34.34 (-162.68, 231.37)**	Moderate	c
MAG vs SEL (Network MA)		**-16.37 (-226.59, 193.85)**	Moderate	c
MAG vs SOY (Network MA)		**54.90 (-162.51, 272.31)**	Moderate	c
MAG vs VIT D (Network MA)		**-32.75 (-239.85, 174.36)**	Moderate	c
MAG vs VIT D – CAL (Network MA)		**-77.32 (-292.07, 137.43)**	Moderate	c
MAG vs ZINC (Network MA)		**26.50 (-184.97, 237.97)**	Moderate	c
MAG vs Ω3 (Network MA)		**-24.87 (-220.34, 170.59)**	Moderate	c
MAG vs Ω3 – VIT D (Network MA)		**4.25 (-199.63, 208.13)**	Moderate	c
MAG vs Ω3 – VIT E (Network MA)		**158.10 (-65.49, 381.69)**	Moderate	c
PROB vs SEL (Network MA)		**-50.71 (-185.67, 84.24)**	Moderate	c
PROB vs SOY (Network MA)		**20.56 (-125.34, 166.45)**	Moderate	c
PROB vs VIT D (Network MA)		**-67.09 (-197.14, 62.96)**	Moderate	c
PROB vs VIT D - CAL (Network MA)		**-111.66 (-253.57, 30.24)**	Moderate	c
PROB vs ZINC (Network MA)		**-7.84 (-144.73, 129.04)**	Moderate	c
PROB vs Ω3 (Network MA)		**-59.22 (-169.79, 51.36)**	Moderate	c
PROB vs Ω3 – VIT D (Network MA)		**-30.09 (-154.94, 94.76)**	Moderate	c
PROB vs Ω3 – VIT E (Network MA)		**123.76 (-31.20, 278.71)**	Moderate	c
SEL vs SOY (Network MA)		**71.27 (-92.01, 234.55)**	Moderate	c
SEL vs VIT D (Network MA)		**-16.38 (-165.67, 132.92)**	Moderate	c
SEL vs VIT D - CAL (Network MA)		**-60.95 (-220.67, 98.77)**	Moderate	c
SEL vs ZINC (Network MA)		**42.87 (-112.41, 198.15)**	Moderate	c
SEL vs Ω3 (Network MA)		**-8.50 (-141.17, 124.17)**	Moderate	c

Outcomes	№ of participants (studies)	Mean Difference (95% CI)	Certainty of the evidence (GRADE)	Comments
SEL vs Ω3 – VIT D (Network MA)		**20.62 (-124.16, 165.41)**	Moderate	c
SEL vs Ω3 – VIT E (Network MA)		**174.47 (3.05,345.89)**	High	
SOY vs VIT D (Network MA)		**-87.65 (-246.90, 71.61)**	Moderate	c
SOY vs VIT D - CAL (Network MA)		**-132.22 (-301.29, 36.85)**	Moderate	c
SOY vs ZINC (Network MA)		**-28.40 (-193.28, 136.48)**	Moderate	c
SOY vs Ω3 (Network MA)		**-79.77 (-223.56, 64.02)**	Moderate	c
SOY vs Ω3 – VIT D (Network MA)		**-50.65 (-205.68, 104.39)**	Moderate	c
SOY vs Ω3 – VIT E (Network MA)		**103.20 (-76.97, 283.37)**	Moderate	c
VIT D vs VIT D - CAL (Network MA)		**-44.57 (-200.18, 111.03)**	Moderate	c
VIT D vs ZINC (Network MA)		**59.25 (-91.80, 210.29)**	Moderate	c
VIT D vs Ω3 – VIT E (Network MA)		**190.85 (23.25, 358.44)**	High	
VIT D – CAL vs ZINC (Network MA)		**103.82 (-57.54, 265.18)**	Moderate	c
VIT D – CAL vs Ω3 (Network MA)		**52.45 (-87.29, 192.18)**	Moderate	c
VIT D – CAL vs Ω3 – VIT D (Network MA)		**81.57 (-69.71, 232.86)**	Moderate	c
VIT D – CAL vs Ω3 – VIT E (Network MA)		**235.42 (58.47,412.37)**	High	
ZINC vs Ω3 (Network MA)		**-51.37 (-186.01, 83.26)**	Moderate	c
ZINC vs Ω3 – VIT D (Network MA)		**-22.25 (-168.83, 124.34)**	Moderate	c
ZINC vs Ω3 – VIT E (Network MA)		**131.60 (-41.35, 304.55)**	Moderate	c
Ω3 vs Ω3 – VIT E (Network MA)		**182.97 (30.00,335.95)**	High	
Ω3 VIT D vs Ω3 – VIT E (Network MA)		**153.85 (-9.74, 317.43)**	Moderate	c

- 1 Downgraded one level for study limitations (≥ 1 studies were at high risk of bias or in case of a single study there were some concerns)
- 4 Downgraded one level for imprecision (sparse data)
- a Downgraded one level for study limitations (>50% of studies were at moderate or high risk of bias)
- c Downgraded one level for imprecision (95% CI for MD includes 0)

Table S4. GRADE of the ΔMDA (changes in the Malonaldehyde) outcome.

Outcomes	№ of participants (studies)	Mean Difference (95% CI)	Certainty of the evidence (GRADE)	Comments
MAG-ZIN-CALvs Placebo (direct evidence)	60 (1 study)	**-0.60 (1.01, -0.19)**	High	
MAG-ZIN-CALvs Placebo (Network MA)		**-0.60 (-1.01,-0.19)**	Moderate	c
MAG vs Placebo (direct evidence)	70 (1 study)	**-0.80 (-1.46, -0.14)**	Low	1,4
MAG vs Placebo (Network MA)		**-0.80 (-1.46,-0.14)**	Low	a, c
PROB vs Placebo (direct evidence)	120 (2 studies)	**-0.45 (-0.74, -0.16)**	High	
PROB vs Placebo (Network MA)		**-0.59 (-1.17 ,0.00)**	High	
SOY vs Placebo (direct evidence)	68 (1 study)	**-0.50 (-1.04, 0.04)**	High	
SOY vs Placebo (Network MA)		**-0.50 (-1.04,0.04)**	Moderate	c
VIT D vs Placebo (direct evidence)	60 (1 study)	**-0.70 (-1.23, -0.17)**	Moderate	4
VIT D vs Placebo (Network MA)		**-0.74 (-1.22,-0.25)**	Moderate	c
VIT D-CAL vs Placebo (direct evidence)	56 (1 study)	**-0.87 (-1.65, -0.09)**	Moderate	4
VIT D-CAL vs Placebo (Network MA)		**0.87 (-1.65,-0.09)**	Moderate	c
ZINC vs Placebo (direct evidence)	50 (1 study)	**-0.90 (-1.83, 0.03)**	High	
ZINC vs Placebo (Network MA)		**-0.90 (-1.83,0.03)**	Very low	c
Ω3 vs Placebo (direct evidence)	114 (2 studies)	**-0.85 (-1.36, -0.34)**	Moderate	4
Ω3 vs Placebo (Network MA)		**-0.85 (-1.36,-0.34)**	High	
Ω3-VIT D vs Placebo (direct evidence)	60 (1 study)	**-1.00 [-1.53, -0.47]**	High	
Ω3-VIT D vs Placebo (Network MA)		**-1.04 (-2.88,0.81)**	Moderate	c
Ω3-VIT E vs Placebo (direct evidence)	60 (1 study)	**-0.70 [-1.30, -0.10]**	High	
Ω3-VIT E vs Placebo (Network MA)		**-0.70 (-1.30,-0.10)**	High	
Ω3 vs VIT D (direct evidence)	60 (1 study)	**33.30 (-22.57,89.12)**	Moderate	4
Ω3 vs VIT D (Network MA)		**-0.12 (-0.47,0.24)**	Moderate	c
Ω3-VIT D vs VIT D (direct evidence)	60 (1 study)	**37.0 (-13.84,87.84)**	Moderate	4
Ω3-VIT D vs VIT D (Network MA)		**-0.30 (-2.10,1.50)**	Moderate	c
Ω3-VIT D vs Ω3 (direct evidence)	60 (1 study)	**3.70 (-38.60,46.0)**	Moderate	4
Ω3-VIT D vs Ω3 (Network MA)		**-0.18 (-2.00,1.63)**	Moderate	c
MAG-ZIN-CALvs MAG (Network MA)		**-0.20 (-0.98,0.58)**	Moderate	c
MAG-ZIN-CALvs PROB (Network MA)		**0.01 (-0.70,0.73)**	Moderate	c
MAG-ZIN-CALvs SOY (Network MA)		**0.10 (-0.58,0.78)**	Moderate	c

Outcomes	№ of participants (studies)	Mean Difference (95% CI)	Certainty of the evidence (GRADE)	Comments
MAG-ZIN-CALvs VIT D (Network MA)		**-0.14 (-0.77,0.50)**	Moderate	c
MAG-ZIN-CALvs VIT D - CAL (Network MA)		**-0.27 (-1.15,0.61)**	Moderate	c
MAG-ZIN-CALvs ZINC (Network MA)		**-0.30 (-1.31,0.71)**	Moderate	c
MAG-ZIN-CALvs Ω3 (Network MA)		**-0.30 (-1.31,0.71)**	Moderate	c
MAG-ZIN-CALvs Ω3 – VIT D (Network MA)		**-0.44 (-2.33,1.45)**	Moderate	c
MAG-ZIN-CALvs Ω3 – VIT E (Network MA)		**-0.10 (-0.82,0.62)**	Moderate	c
MAG vs PROB (Network MA)		**0.21 (-0.67,1.10)**	Moderate	c
MAG vs SOY (Network MA)		**0.30 (-0.56,1.16)**	Moderate	c
MAG vs VIT D (Network MA)		**0.06 (-0.76,0.88)**	Moderate	c
MAG vs VIT D – CAL (Network MA)		**--0.07 (-1.09,0.95)**	Moderate	c
MAG vs ZINC (Network MA)		**-0.10 (-1.24,1.04)**	Moderate	c
MAG vs Ω3 (Network MA)		**-0.05 (-0.89,0.78)**	Moderate	c
MAG vs Ω3 – VIT D (Network MA)		**-0.24 (-2.20,1.72)**	Moderate	c
MAG vs Ω3 – VIT E (Network MA)		**0.10 (-0.79,0.99)**	Moderate	c
PROB vs SOY (Network MA)		**0.09 (-0.71,0.88)**	Moderate	c
PROB vs VIT D (Network MA)		**-0.15 (-0.91,0.61)**	Moderate	c
PROB vs VIT D - CAL (Network MA)		**-0.28 (-1.26,0.69)**	Moderate	c
PROB vs ZINC (Network MA)		**-0.31 (-1.41,0.78)**	Moderate	c
PROB vs Ω3 (Network MA)		**-0.27 (-1.04,0.51)**	Moderate	c
PROB vs Ω3 – VIT D (Network MA)		**-0.45 (-2.39,1.48)**	Moderate	c
PROB vs Ω3 – VIT E (Network MA)		**-0.11 (-0.95,0.72)**	Moderate	c
SOY vs VIT D (Network MA)		**-0.24 (-0.97,0.49)**	Moderate	c
SOY vs VIT D - CAL (Network MA)		**-0.37 (-1.32,0.58)**	Moderate	c
SOY vs ZINC (Network MA)		**-0.40 (-1.47,0.67)**	Moderate	c
SOY vs Ω3 (Network MA)		**-0.35 (-1.10,0.39)**	Moderate	c
SOY vs Ω3 – VIT D (Network MA)		**-0.54 (-2.46,1.39)**	Moderate	c
SOY vs Ω3 – VIT E (Network MA)		**-0.20 (-1.01,0.61)**	Moderate	c
VIT D vs VIT D - CAL (Network MA)		**-0.13 (-1.05,0.79)**	Moderate	c
VIT D vs ZINC (Network MA)		**-0.16 (-1.21,0.88)**	Moderate	c
VIT D vs Ω3 (Network MA)		**-0.12 (-0.47,0.24)**		
VIT D vs Ω3– VIT D (Network MA)		**-0.30 (-2.10,1.50)**		

Outcomes	№ of participants (studies)	Mean Difference (95% CI)	Certainty of the evidence (GRADE)	Comments
VIT D vs Ω3 – VIT E (Network MA)		**0.04 (-0.73,0.81)**	High	
VIT D – CAL vs ZINC (Network MA)		**-0.03 (-1.24,1.18)**	Moderate	c
VIT D – CAL vs Ω3 (Network MA)		**0.02 (-0.92,0.95)**	Moderate	c
VIT D – CAL vs Ω3 – VIT D (Network MA)		**-0.17 (-2.17,1.84)**	Moderate	c
VIT D – CAL vs Ω3 – VIT E (Network MA)		**0.17 (-0.81,1.15)**	High	
ZINC vs Ω3 (Network MA)		**0.05 (-1.01,1.10)**	Moderate	c
ZINC vs Ω3 – VIT D (Network MA)		**-0.14 (-2.20,1.93)**	Moderate	c
ZINC vs Ω3 – VIT E (Network MA)		**0.20 (-0.90,1.30)**	Moderate	c
Ω3 vs Ω3 – VIT D (Network MA)		**-0.18 (-2.00,1.63)**		
Ω3 vs Ω3 – VIT E (Network MA)		**0.15 (-0.63,0.94)**	High	
Ω3 VIT D vs Ω3 – VIT E (Network MA)		**0.34 (-1.60,2.28**	Moderate	c

- 1 Downgraded one level for study limitations (≥ 1 studies were at high risk of bias or in case of a single study there were some concerns)
- 4 Downgraded one level for imprecision (sparse data)
- a Downgraded one level for study limitations (>50% of studies were at moderate or high risk of bias)
- c Downgraded one level for imprecision (95% CI for MD includes 0)

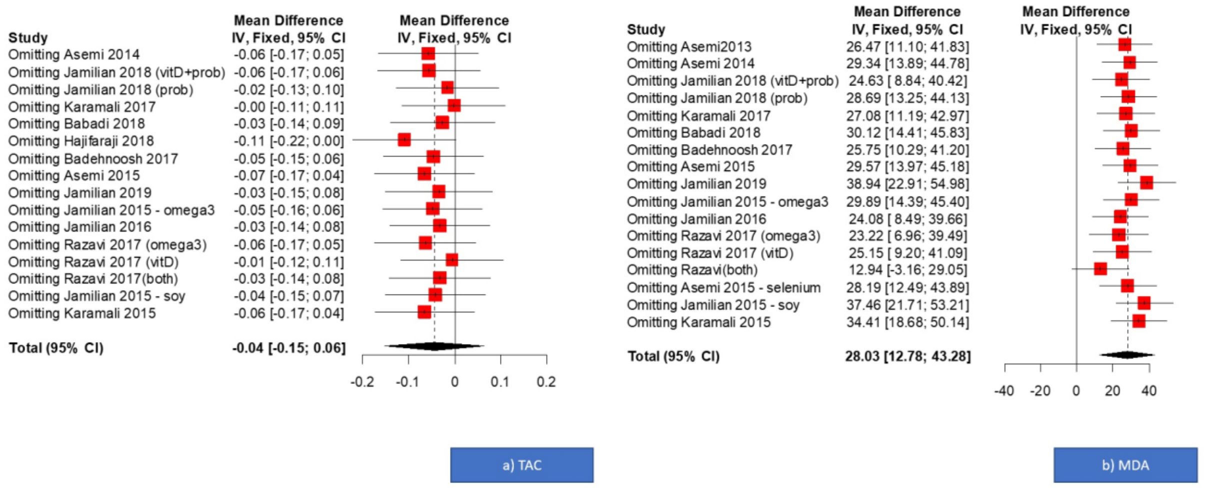

Figure S1: Leave one out analysis for the baseline values of A) TAC and B) MDA, investigating the influence of each individual study on the overall meta-analysis summary estimate.

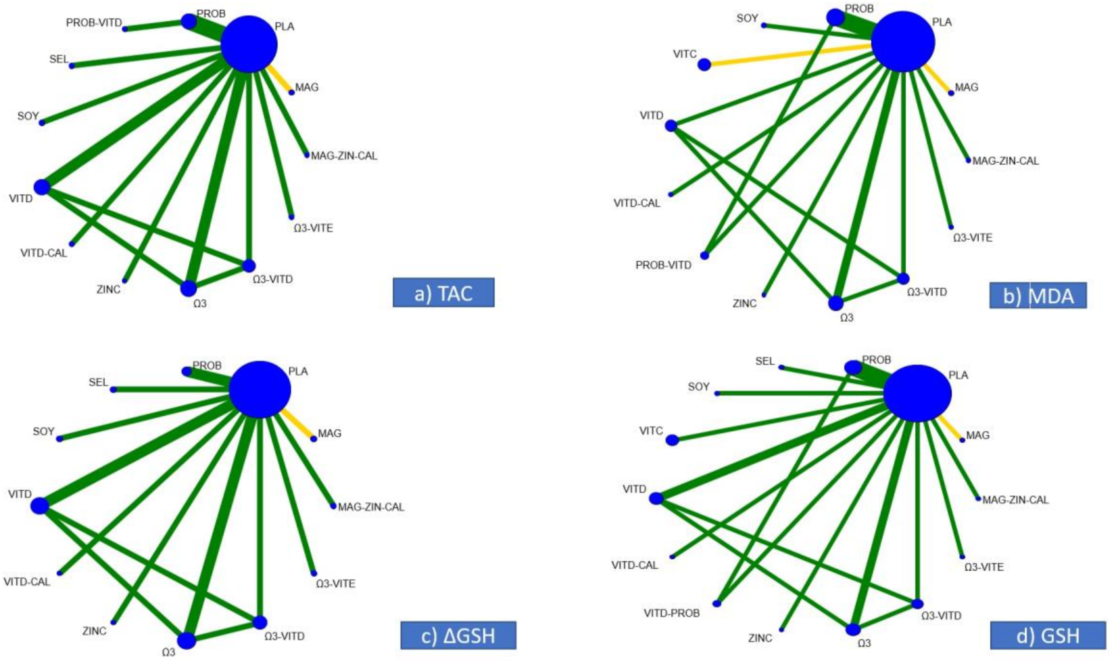

Figure S2: Network plots for the secondary outcomes of A) TAC B) MDA, C) ΔGSH, D) GSH. Treatments are represented by nodes and head-to-head comparisons with edges. The size of the nodes is proportional to the number of the patients, while the thickness of the edges is proportional to the number of studies. The color of the edges represents the average risk of bias for each head-to-head comparison, green for low risk of bias, yellow for uncertain risk of bias, and red for high risk of bias.

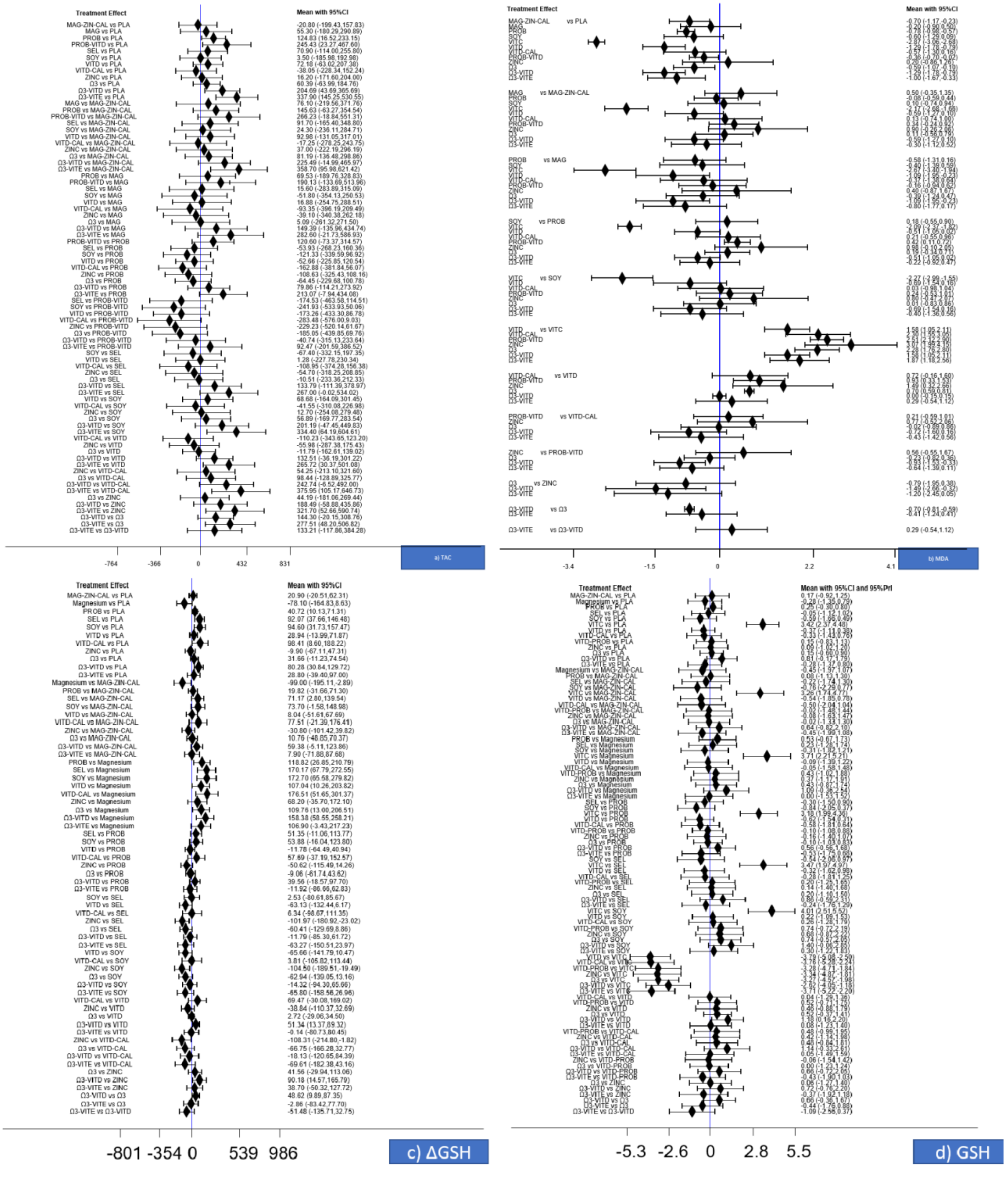

Figure S3: Mean difference (MD) for A) TAC, B) MDA, C) ΔGSH and D) GSH as estimated from the network meta-analysis for every possible pair of interventions. Solid lines represent 95% Confidence Intervals (Cis)

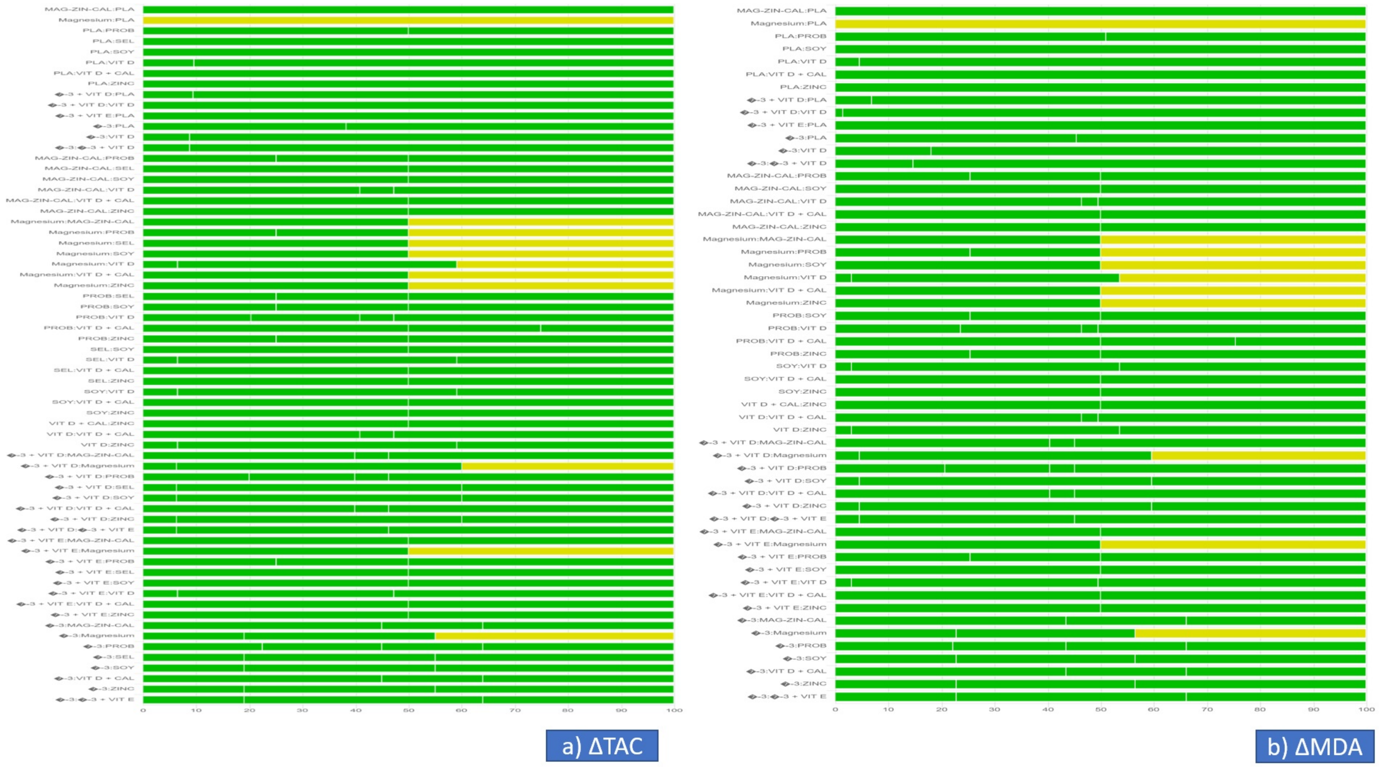

Figure S4: The contributions of direct and indirect data to the network estimate.

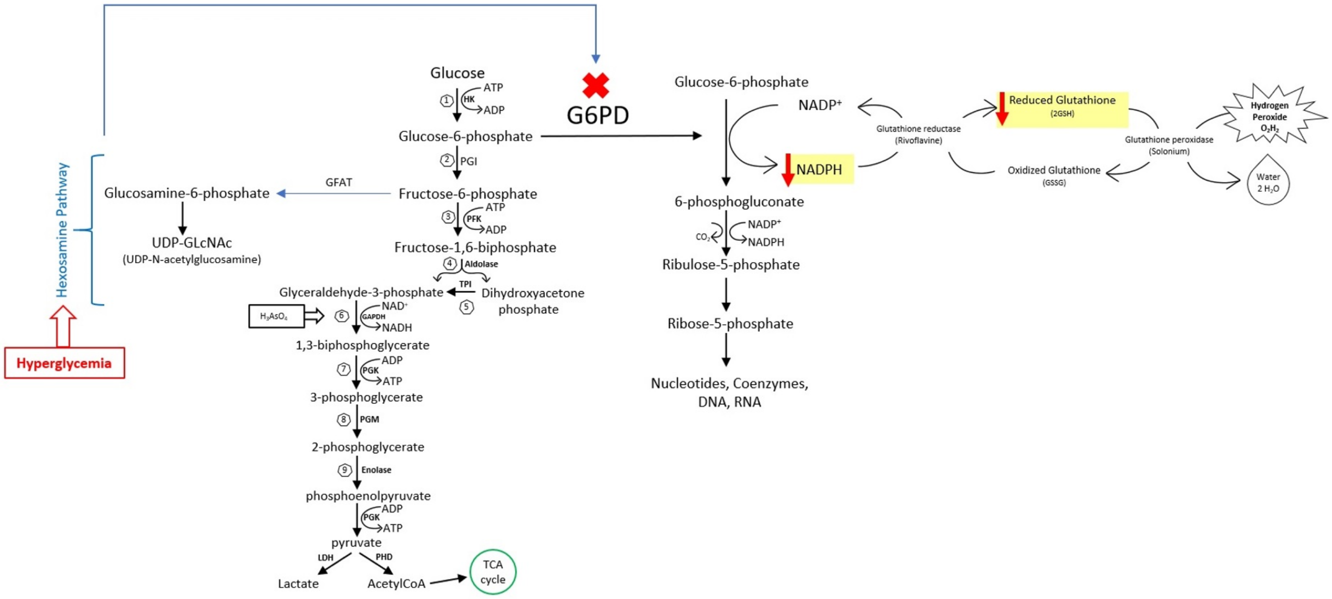

Figure S5: Pathophysiology between hyperglycemia and impairment of antioxidant mechanisms
